# ZEB2 drives immature T-cell lymphoblastic leukaemia development via enhanced tumour-initiating potential and IL-7 receptor signalling

**DOI:** 10.1038/ncomms6794

**Published:** 2015-01-07

**Authors:** Steven Goossens, Enrico Radaelli, Odile Blanchet, Kaat Durinck, Joni Van der Meulen, Sofie Peirs, Tom Taghon, Cedric S. Tremblay, Magdaline Costa, Morvarid Farhang Ghahremani, Jelle De Medts, Sonia Bartunkova, Katharina Haigh, Claire Schwab, Natalie Farla, Tim Pieters, Filip Matthijssens, Nadine Van Roy, J. Adam Best, Kim Deswarte, Pieter Bogaert, Catherine Carmichael, Adam Rickard, Santi Suryani, Lauryn S. Bracken, Raed Alserihi, Kirsten Canté-Barrett, Lieven Haenebalcke, Emmanuelle Clappier, Pieter Rondou, Karolina Slowicka, Danny Huylebroeck, Ananda W. Goldrath, Viktor Janzen, Matthew P. McCormack, Richard B. Lock, David J. Curtis, Christine Harrison, Geert Berx, Frank Speleman, Jules P. P. Meijerink, Jean Soulier, Pieter Van Vlierberghe, Jody J. Haigh

**Affiliations:** 1 Vascular Cell Biology Unit, VIB Inflammation Research Center, Ghent University, Ghent B-9052, Belgium; 2 Department for Biomedical Molecular Biology, Ghent University, Ghent B-9052, Belgium; 3 Unit for Molecular and Cellular Oncology, VIB Inflammation Research Center, Ghent University, Ghent B-9052, Belgium; 4 Mammalian Functional Genetics Laboratory, Division of Blood Cancers, Australian Centre for Blood Diseases, Department of Clinical Haematology, Monash University and Alfred Health Alfred Centre, 99 Commercial Road, Melbourne, Victoria 3004, Australia; 5 Mouse and Animal Pathology Laboratory, Department of Veterinary Medicine, Università degli Studi di Milano, Milan 20122, Italy; 6 VIB11 Center for the Biology of Disease, KU Leuven Center for Human Genetics, KU Leuven, Leuven B-3000, Belgium; 7 Institut Universitaire d’Hématologie and U944 INSERM, Hopital Saint-Louis, Paris 75010, France; 8 Department of Hematology and Immunology, CHU, Angers 49100, France; 9 Center for Medical Genetics, Ghent University Hospital, Ghent B-9000, Belgium; 10 Department of Clinical Chemistry, Microbiology and Immunology, Ghent University, Ghent B-9000, Belgium; 11 Stem Cell Research Group, Division of Blood Cancers, Australian Centre for Blood Diseases, Monash University, Melbourne, Victoria 3004, Australia; 12 Northern Institute for Cancer Research, Newcastle University, Newcastle-upon-Tyne NE2 4AD, UK; 13 Division of Biological Sciences, Section of Molecular Biology, University of California at San Diego, La Jolla, California 92093, USA; 14 VIB Inflammation Research Center, Ghent University, Ghent B-9052, Belgium; 15 Children’s Cancer Institute, Leukaemia Biology Program, Lowy Cancer Research Centre, University of New South Wales, Sydney, New South Wales 2052, Australia; 16 Cancer and Haematology Division, Walter and Eliza Hall Institute of Medical Research, Melbourne University, Parkville, Victoria 3052, Australia; 17 Department of Pediatric Oncology/Hematology, Erasmus MC Rotterdam—Sophia Children’s Hospital, Rotterdam 3015CE, The Netherlands; 18 Department of Development and Regeneration, KU Leuven, Leuven B-3000, Belgium; 19 Department of Cell Biology, Erasmus MC, Rotterdam 3015CE, The Netherlands; 20 Department of Internal Medicine III, Hematology and Oncology, University of Bonn, D-53012 Bonn, Germany

## Abstract

Early T-cell precursor leukaemia (ETP-ALL) is a high-risk subtype of human leukaemia that is poorly understood at the molecular level. Here we report translocations targeting the zinc finger E-box-binding transcription factor *ZEB2* as a recurrent genetic lesion in immature/ETP-ALL. Using a conditional gain-of-function mouse model, we demonstrate that sustained *Zeb2* expression initiates T-cell leukaemia. Moreover, *Zeb2*-driven mouse leukaemia exhibit some features of the human immature/ETP-ALL gene expression signature, as well as an enhanced leukaemia-initiation potential and activated Janus kinase (JAK)/signal transducers and activators of transcription (STAT) signalling through transcriptional activation of *IL7R*. This study reveals ZEB2 as an oncogene in the biology of immature/ETP-ALL and paves the way towards pre-clinical studies of novel compounds for the treatment of this aggressive subtype of human T-ALL using our *Zeb2*-driven mouse model.

## Abstract (web summary)

Driver mutations in early T-cell precursor leukaemia (ETP-ALL) are poorly characterized. Here the authors show that *Zeb2* overexpression is often found in ETP-ALL, can recapitulate the disease in transgenic mice and confers survival advantage by upregulating IL-7 signalling.

## Graphical abstract

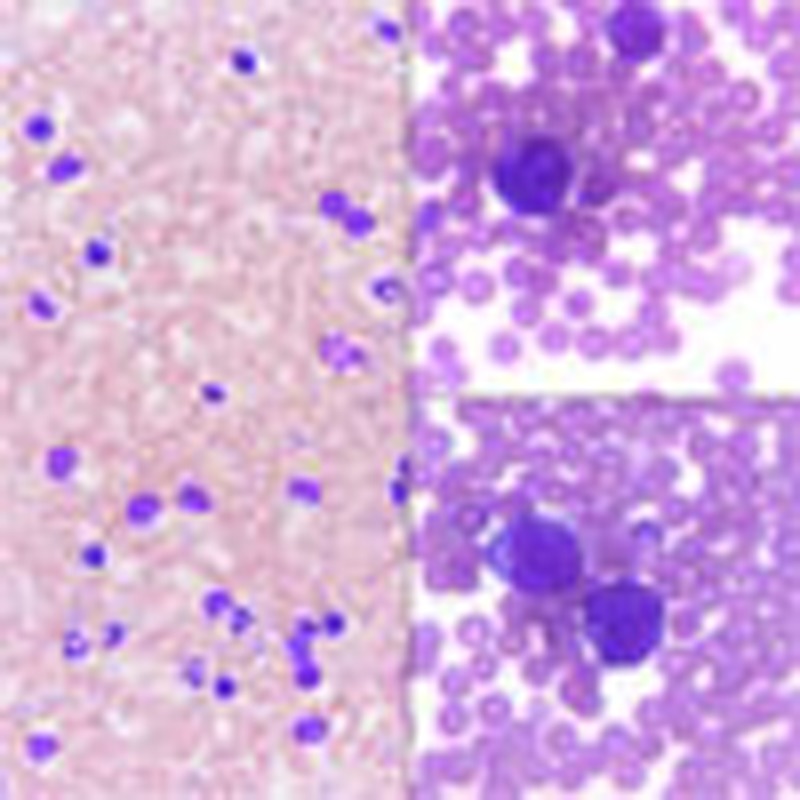

T-cell acute lymphoblastic leukaemia (T-ALL) arises due to multiple genetic lesions in immature T cells leading to a block in differentiation, increased survival and clonal proliferative expansion of a malignant clone^1^,^2^. Despite the improved survival rates because of intensified chemotherapy and bone marrow transplantation, 20% of T-ALL patients still relapse with little or no perspective for cure.

One particularly challenging subgroup of T-ALL is the LYL1^+^/immature/early T-cell precursor leukaemia (ETP-ALL)^3^,^4^. Lymphoblasts of these patients exhibit gene expression profiles similar to ETPs^5^,^6^, the most primitive T-cell progenitor cells within the thymus. Although it has been recognized that a high mutation load characterizes this heterogeneous subgroup^7^, the nature of the driving oncogenic events remains largely unknown. Strikingly, these immature leukaemia patients show inferior survival rates as compared with the other T-ALL subgroups^5^,^7^. The basis of this poor prognosis has yet to be identified, although one potential contributing factor could reside in enhanced leukaemic stem cell properties of this aggressive T-ALL subtype.

Here we report the identification of a recurrent t(2;14)(q22;q32) translocation targeting the human *ZEB2* locus in immature/ETP-ALL, indicating that deregulation of *ZEB2* expression can act as an initiating driver event for this aggressive disease. ZEB2 is a member of the Zinc finger E-box-binding family of transcription factors^8^,^9^, whose altered expression has previously been correlated with the acquisition of cancer stem cell properties^10^,^11^. Using a conditional *Zeb2* gain-of-function mouse model, we provide further evidence for a key oncogenic role of ZEB2 in the biology of immature/ETP-ALL driven by enhanced stem cell characteristics and activation of the IL7R-JAK/signal transducers and activators of transcription (STAT) signalling pathway.

## Results

### *ZEB2* locus translocations in human immature/ETP-ALL

**Figure 1:**
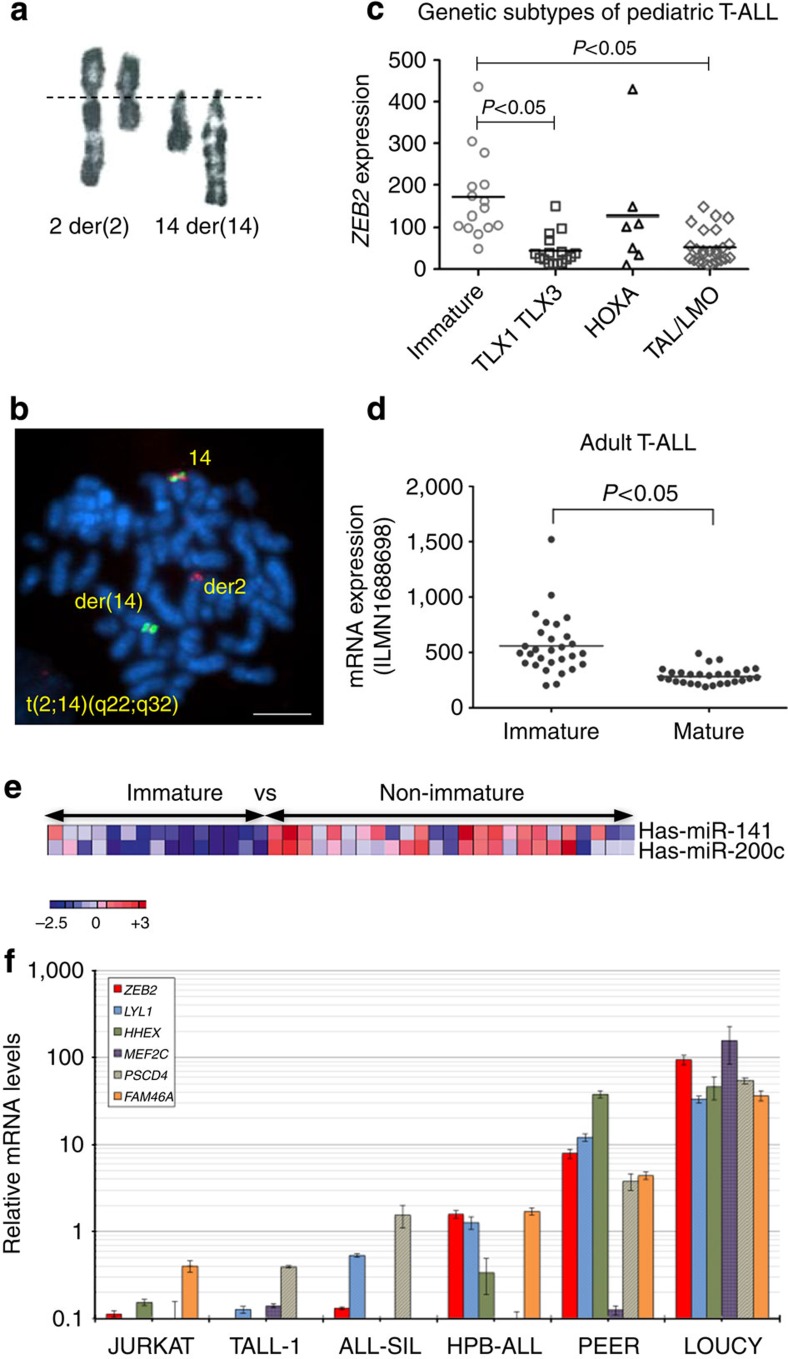
Identification of ETP-ALL cases with translocations involving the *ZEB2* locus and enhanced *ZEB2* expression in the immature subtype of human T-ALL patients. (**a**) Partial karyotype (RHG banding) in case TL88 showing a t(2;14)(q22;q32) translocation. (**b**) Dual-colour FISH analysis using combination of BCL11B and ZEB2 probes on metaphase spreads from leukaemic cells in case TL88. FISH probe RP11-68I8 is shown in green, RP11-889B13 in red. Scale bar, 10 μm. (**c**) *ZEB2* expression levels in a previously published^4^ cohort of 64 human paediatric T-ALL cases. Unsupervised hierarchical classification of T-ALL subtypes was based on gene expression and genomic annotations. Mean expression is indicated and Mann–Whitney *U*-Test used for statistical analysis. (**d**) *ZEB2* expression levels in an independent published cohort of 57 human adult T-ALL cases. Unsupervised hierarchical classification of immature and mature subclasses was based on gene expression and genomic annotations as reported^17^. Mean expression is indicated and Mann–Whitney *U*-Test used for statistical analysis. (**e**) Heatmap showing expression levels of the miRNA-141/200c cluster in a published cohort of human T-ALL patients^48^. (**f**) qRT–PCR analysis; relative mRNA expression for *ZEB2* and the immature marker genes *LYL1*, *HHEX*, *MEF2C*, *PSCD4*, *FAM46A* in six human T-ALL cell lines. Data were presented as average±s.d.

Karyotyping of T-ALL samples revealed a novel chromosomal translocation t(2;14)(q22;q32) (Fig. 1a) in two patients with a typical immature/ETP-ALL immunophenotype^5^. Interestingly, the *BCL11B* gene resides in chromosome band 14q32 and given its implication in translocations leading to overexpression of several *bona fide* T-ALL oncogenes, such as *HOX11L2/TLX3, HOXA13* (refs 12, 13) and *NKX2-5* (refs 14, 15), we performed fluorescence *in situ* hybridization (FISH) analysis using locus-specific probes. This confirmed the disruption of the *BCL11B* locus at 14q32 and suggested the presence of a novel ETP-ALL oncogene located at 2q22 in these two immature/ETP-ALL patients. In order to identify the presumed oncogene, subsequent breakpoint mapping was performed using FISH probes within chromosome band 2q22, allowing us to map the breakpoint within the vicinity of the *ZEB2* locus (Fig. 1b). Next, we screened an independent T-ALL cohort, comprising of 1,084 patients and identified two additional T-ALL patient samples with an identical t(2;14)(q22;q32) translocation. Taken together, we identified a novel and rare, but recurrent translocation involving *ZEB2* and *BCL11B* in human T-ALL.

### *ZEB2* levels are elevated in human immature/ETP-ALL

Microarray-based genome-wide expression studies have shown that T-ALL encompasses distinct molecular groups with defined gene expression signatures. Several key genes, such as *LYL1*, *MEF2C* and *HHEX*, show elevated levels of gene expression in the immature/ETP-ALL subclass^3^,^4^,^5^,^6^ as compared with other T-ALL subtypes. Gene expression profiling data were available for one of the four t(2;14)(q22;q32) cases (TL88)^4^, and showed an immature/ETP-ALL gene signature. *ZEB2* mRNA levels in this particular patient were increased as compared with mature T-ALL subclasses, but within the same range to other immature/ETP-ALL patients without t(2;14)(q22;q32) translocations (Supplementary Fig. 1a). *BCL11B* levels in the TL88 sample were not significantly lower as compared with other immature/ETP-ALL samples (Supplementary Fig. 1b).

Analysis of gene expression profiles from paediatric^16^ and adult^17^ T-ALL patient series confirmed that *ZEB2* expression levels were predominantly higher (Fig. 1c,d), and inversely correlated with the lower levels of miR200c (Fig. 1e) within immature/ETP-ALL patients.

Moreover, real-time quantitative PCR (qRT–PCR) analysis confirmed high *ZEB2* expression in LOUCY cells (Fig. 1f), a T-ALL cell line with a transcriptional signature highly similar to that of immature/ETP-ALL patients^6^,^17^ (Supplementary Fig. 2).

Taken together, the observed recurrent t(2;14)(q22;q34) translocations involving the *ZEB2* locus in combination with high *ZEB2* levels throughout human immature/ETP-ALL, suggest that ZEB2 might act as an oncogenic driver in this subset of human T-ALL.

### Increased *Zeb2* expression results in T-cell leukaemia in mice

**Figure 2:**
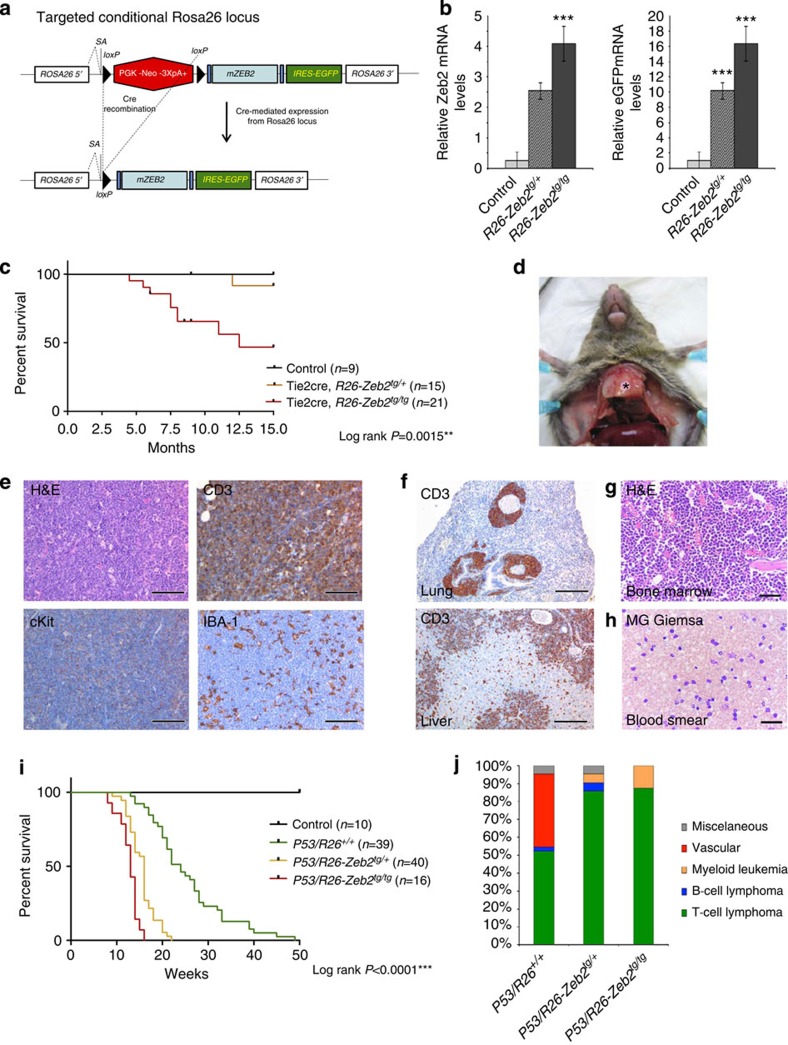
Tie2-Cre-mediated expression of *Zeb2* on its own or in synergy with *p53* loss leads to T-cell lymophoblastic leukaemia. (**a**) Schematic representation of the conditional *ROSA26*-based *Zeb2* overexpression mouse model. The mouse *Zeb2* open reading frame was targeted to the *ROSA26* locus preceded by a floxed (fl) transcriptional stop (PGK-Neo-3XpA) cassette and followed by IRES-EGFP reporter sequence. (**b**) Quantification of relative Zeb2 and EGFP mRNA levels in thymus of *Tie2cre, R26-Zeb2*^*tg/+*^ (*R26-Zeb2*^*tg/+*^) and *Tie2cre, R26-Zeb2*^*tg/tg*^ (*R26-Zeb2*^*tg/tg*^) mice compared with Tie2-Cre-negative control littermates (Control). qRT–PCR data were presented as average±s.d. (**c**) Kaplan–Meier survival curve of *R26-Zeb2*^*tg/tg*^ mice (*n*=21) compared with control littermates (*n*=9). Mantel–Cox test was used for statistical analysis. (**d**) From 5 months of age, *R26-Zeb2*^*tg/tg*^ spontaneously develop thymus tumours (asterisk). (**e**) Histopathological analysis of thymic mass displaying dense monomorphic sheets of neoplastic lymphoid cells; haematoxylin and eosin (H&E) staining, scale bar, 100 μm. Neoplastic lymphoid cells are diffusely positive for the T-cell marker CD3; CD3 immunohistochemistry, scale bar, 80 μm. Neoplastic T cells also exhibit moderate degree of cKit expression; cKit immunohistochemistry, scale bar, 100 μm. IBA-1-positive histiocytes/macrophages are scattered among the neoplastic lymphoid sheets; IBA-1 immunohistochemistry, scale bar, 100 μm. (**f**) Precursor T-cell lymphoblastic leukaemia (Pre-T LBL) invading the adjacent pulmonary parenchyma with dense infiltrates of CD3-positive cells expanding the peribronchial/perivascular interstitium; CD3 immunohistochemistry, scale bar, 400 μm. PTLL exhibits systemic dissemination with dense infiltrates of CD3-positive cells expanding the centrilobular and portal areas and dissecting along the surrounding hepatic cords; CD3 immunohistochemistry, scale bar, 200 μm. (**g**) Bone marrow of *R26-Zeb2*^*tg/tg*^ with Pre-T LBL. H&E stain, scale bar, 50 μm. (**h**) Microscopic analysis of blood smears from *R26-Zeb2*^*tg/tg*^ with Pre-T LBL, with a high number of large and atypical leukaemic lymphoid cells in the circulation. May Grunwald Giemsa stain, scale bar, 50 μm. (**i**) Kaplan–Meier survival curve after intercrossing *Zeb2*-overexpressing mice on to a *Tie2cre, p53*^*f/f*^ thymic tumour-prone background: a significant decrease in tumour latency and survival could be observed (**j**) with a shift in tumour spectrum. Mantel–Cox test was used to determine significance for Kaplan–Meier curve. **P<0.01, ***P<0.001.

To further study the role of ZEB2 as a putative oncogene in the pathogenesis of T-ALL, we crossed our recently developed conditional ROSA26-based *Zeb2* gain-of-function mouse model^18^ with the Tie2-Cre line^19^. Upon Cre expression and removal of the floxed stop cassette (Fig. 2a) in all endothelial and haematopoietic cells^19^,^20^, a bicistronic transcript encoding ZEB2 and the enhanced green fluorescent protein reporter (EGFP) is expressed from the *ROSA26* promoter. Heterozygous *Zeb2* overexpression (*R26-Zeb2*^*tg/+*^) resulted in twofold upregulation of the total thymic *Zeb2* mRNA levels (Fig. 2b). Homozygous *R26-Zeb2*^*tg/tg*^ mice displayed a doubling of transgene expression leading to a three- to fourfold *Zeb2* mRNA upregulation (Fig. 2b).

*R26-Zeb2*^*tg/tg*^ mice (*n*=21) can survive until adulthood and peripheral blood analysis from 6- to 8-week-old mice revealed no significant difference in blood cell composition at this age (Supplementary Fig. 3). *R26-Zeb2*^*tg/tg*^ mice, however, start to die from 5 months of age onwards, with 53% of these mice dying by 15 months of age (Fig. 2c). At autopsy, mediastinal masses were detected (Fig. 2d). Detailed pathological examination diagnosed these mice with precursor T-cell lymphoblastic leukaemia (Pre-T LBL) as determined by immunohistochemistry: CD45/CLA^+^; CD3^+^; CD45/B220^−^ and IBA-1^−^ with the presence of cKit^+^ cells (Fig. 2e). The Pre-T LBL likely originated from the thymus with sheets composed of medium to large-sized lymphoid cells infiltrating into the surrounding tissues, including lung, heart and cranial mediastinum and systemically into lymph nodes, liver, spleen, kidney and bone marrow (Fig. 2f,g). Blood smears detected a high percentage of atypical lymphoid cells in the circulation (Fig. 2h).

To further investigate the oncogenic role of *Zeb2* in the progression of thymic tumours in a more robust manner with increased penetrance, we crossed our Tie2-cre, R26-Zeb2 double transgenic mouse model onto a p53^fl/fl^ background^21^, which is prone to develop thymic tumors. In addition, the p53 model allowed us to further investigate potential synergism with other aberrantly expressed genes in malignant T-cell development. These mice are denoted as control *P53/R26*^*+/+*^ and *Zeb2*-overexpressing *P53/R26-Zeb2*^*tg/+*^ or *P53/R26-Zeb2*^*tg/tg*^ mice/tumours for the remainder of this study. A significant and dosage-dependent decrease in tumour latency (Fig. 2i) and a clear shift in tumour spectrum were observed upon overexpression of *Zeb2* on a p53 knockout background. Control *P53/R26*^*+/+*^ mice developed neoplastic lesions from both vascular (40.5%) and haematopoietic origins (54.8%)^22^, mainly T-cell lymphomas, at an average age of 25.5 weeks (Supplementary Table 1). *P53/R26-Zeb2*^*tg/+*^ or *P53/R26-Zeb2*^*tg/tg*^ mice developed tumours significantly faster, after 15.5 and 12.7 weeks, respectively, and with a complete shift towards haematopoietic neoplastic lesions (100%), mainly precursor T-cell lymphoma (85.7 and 87.5%; Fig. 2j and Supplementary Table 1). No differences were observed in leukaemia distribution and/or systemic organ involvement between *Zeb2*-overexpressing and control mice (data not shown). T-cell antigen receptor rearrangement analysis was suggestive of either mono- or oligoclonal origin for all of the examined thymic tumours irrespective of genotype (Supplementary Fig. 4).

To investigate the presence of cooperating mutations involved in the pathogenesis of *Zeb2*-induced leukaemias, and to determine to what extent this leukaemic mouse model recapitulates human T-ALL, we performed mutation analysis and array Comparative Genomic Hybridization profiling in control (*P53/R26*^*+/+*^) and *Zeb2*-overexpressing tumours (*R26-Zeb2*^*tg/tg*^ and *P53/R26-Zeb2*^*tg/+*^ or *P53/R26-Zeb2*^*tg/tg*^). We identified prototypical *Notch1* mutations (Supplementary Fig. 5a and Supplementary Table 2) as well as focal deletions affecting the *Pten* and *Ikzf1* loci (Supplementary Fig. 5b,c and Supplementary Table 2).

To confirm the autocrine nature of this spontaneously arising lymphoblastic leukaemia, *Zeb2*-overexpressing mice were crossed with a T-cell-restricted CD4-cre line^23^. Here we were able to recapitulate leukaemia development and confirm the T-cell origin of this disease (Supplementary Fig. 6).

### *Zeb2*-expressing thymomas have an immature/ETP-ALL signature

**Figure 3:**
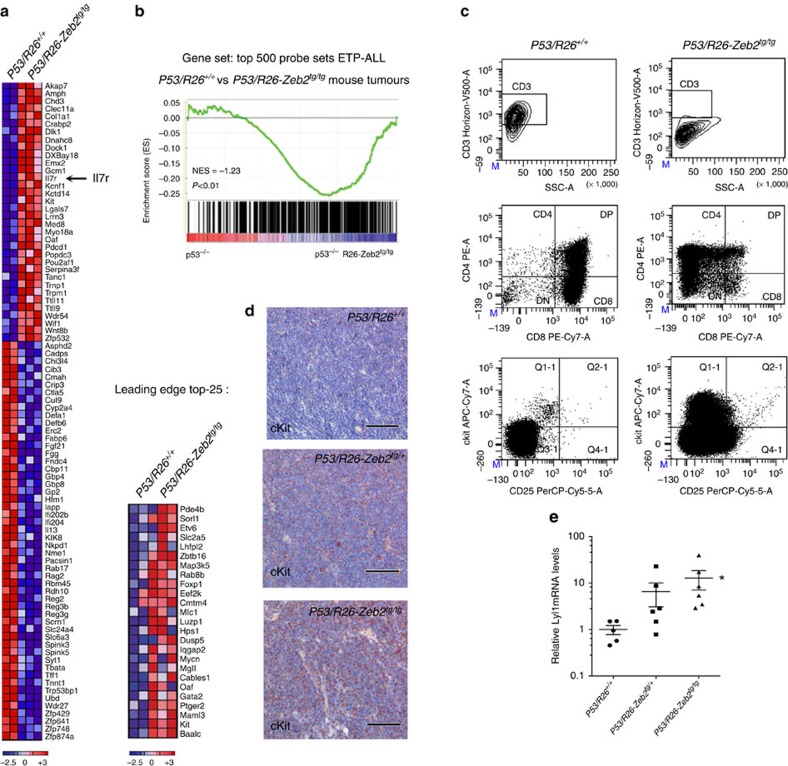
*Zeb2* overexpression leads to increased expression of immature/stem cell marker genes, and ETP-ALL-like expression profile. (**a**) Differential gene expression profiling of *P53/R26*^*+/+*^ (*n*=2) vs *P53/R26-Zeb2*^*tg/tg*^ (*n*=3) thymic tumours (log_2_(FC)>3, *P*<0.05). A heatmap of the top differentially expressed genes is shown. Genes in the heat map are shown in rows and each individual tumour sample is shown in one column. (**b**) Gene set enrichment analysis of a top-500 probe sets enriched in immature/ETP-ALL patients in the gene expression signature of control vs *Zeb2*-overexpressing mouse tumours. The top 25 leading edge genes are also shown. (**c**) Flow cytometric analysis of thymic tumours upon Zeb2 overexpression showing a decrease in more mature markers sCD3 and CD8 and increase of the stem/progenitor marker cKit. Dotplots of representative control P53/R26^+/+^ and *Zeb2*-overexpressing tumours *P53/R26-Zeb2*^*tg/tg*^ are depicted. (**d**) Immunohistochemistry for cKit of representative tumours for each genotype (**e**) qRT–PCR analysis of control *P53/R26*^*+/+*^ and *Zeb2*-overexpressing *P53/R26-Zeb2*^*tg/+*^ and *P53/R26-Zeb2*^*tg/tg*^ thymic tumours for the immature/ETP-ALL marker gene *Lyl1*. Mean expression is indicated±s.d. *P<0.05 (vs control).

Next, we performed differential expression analysis on *P53/R26-Zeb2*^*tg/tg*^ vs control *p53/R26-Zeb2*^*+/+*^ tumours using Agilent microarray hybridization (Fig. 3a). Gene Set Enrichment Analysis using the top-500 probe sets overexpressed in immature/ETP-ALL patients, demonstrated that the gene expression profile of the *Zeb2*-overexpressing mouse tumours significantly overlapped with that of human immature/ETP-ALL patients (Fig. 3b). *Zeb2*-overexpressing thymic tumours lack mature T-cell markers such as surface CD3 and CD8 and exhibited a higher percentage of cells that expressed the stem/progenitor marker cKit or CD44, when compared with the control *P53/R26*^*+/+*^-deficient tumours (Fig. 3c). This increase in cKit^+^ cells in the *Zeb2* overexpression tumours was confirmed via immunohistochemistry (Fig. 3d and Supplementary Table 3). qRT–PCR analysis for the immature marker gene *Lyl1* showed a significant overexpression in our *P53/R26-Zeb2*^*tg/tg*^ tumours compared with the control tumours (Fig. 3e and Supplementary Fig. 7a). A similar trend could be observed for other immature marker genes like *Hhex* and *Mef2c* (Supplementary Fig. 7b). In addition, downregulation of the *Ptcra* (Pre-T-cell Receptor alpha) mRNA levels could be observed upon *Zeb2* overexpression in the p53 null thymic tumours (Supplementary Fig. 7c), similar to what is previously seen in human^5^ and mouse^24^,^25^ immature/ETP-ALL.

Of note, in our control *P53/R26*^*+/+*^ cohort (*n*=39), one mouse spontaneously developed a thymoma with immature T-ALL characteristics; with low tumour latency, increased cKit protein levels as well as high *Lyl1*, *Hhex*, *Mef2c* mRNA expression that all correlated with elevated endogenous *Zeb2* levels (Supplementary Fig. 8). These results suggest that immature ETP-ALL-like T-ALL can develop spontaneously in p53 knockout mice.

### *Zeb2* modulation affects leukaemic cancer stem cell properties

**Figure 4:**
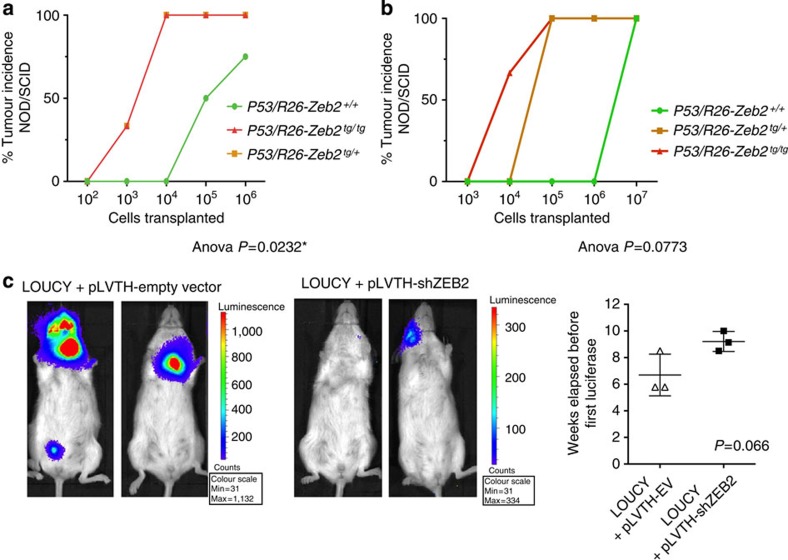
*Zeb2* overexpression leads to increased leukaemia-initiating potential. (**a**) Quantification of leukaemic stem cell (LSC) frequency in control *P53/R26*^*+/+*^ (*n*=4) and *Zeb2*-overexpressing *P53/R26-Zeb2*^*tg/+*^ (*n*=3) and *P53/R26-Zeb2*^*tg/tg*^ (*n*=3) thymic tumours via serial dilution transplantation of primary tumours cells into immunodeficient NOD/SCID recipient mice. One-way analysis of variance (ANOVA) statistical analysis was used. (**b**) Secondary leukaemia-initiating capacity was tested for *P53/R26-Zeb2*^*+/+*^ tumours (*n*=2) vs *P53/R26-Zeb2*^*tg/+*^ (*n*=1) and *P53/R26-Zeb2*^*tg/tg*^ (*n*=4) tumours via serial dilution transplantation of primary tumour cells into NOD/SCID mice. The cell lines were derived from individual mouse thymic tumours and kept in culture for at least six passages. One-way ANOVA statistical analysis was used. (**c**) Transplantation of Luciferase-positive LOUCY cells in NSG mice after ZEB2 knockdown (+pLV-TH shZEB2, *n*=3) resulted in decreased leukaemia development compared with the control cell line (+pLV-TH empty vector, *n*=3). Student’s *t*-test was used for statistical analysis. All leukaemia cell transplantation experiments were conducted once.

The increased expression of *cKit* and *Lyl1* suggested the presence of more leukaemic stem cells upon *Zeb2* overexpression. To further investigate whether there may be differences in leukaemic cancer stem cell properties between *P53/R26*^*+/+*^ control and *P53/R26-Zeb2*^*tg/+*^ or *P53/R26-Zeb2*^*tg/tg*^ tumours, we performed serial dilution tumour transplantation experiments in immunodeficient NOD/SCID mice. This analysis revealed a 60-fold increase in leukaemia-initiating cells in the *P53/R26-Zeb2*^*tg/+*^- and *P53/R26-Zeb2*^*tg/tg*^-overexpressing tumours (0.33 leukaemic stem cell (LSC) per 10^3^ tumour cells) compared with the *P53/R26*^*+/+*^ control tumours (0.50 LSC per 10^5^ tumour cells) (Fig. 4a). Similar differences were obtained by transplanting the derived mouse cell lines, suggesting that the increased leukaemia-initiating capacity is maintained upon *in vitro* culturing (Fig. 4b). In addition, a 65% knockdown of *ZEB2* in the luciferase-positive human immature-like T-ALL cell line LOUCY resulted in decreased tumour formation when transplanted into NOD/SCID/Il2Rγ null recipients (Fig. 4c and Supplementary Fig. 9a,b).

### *Zeb2* overexpression results in increased *Il7r* expression

**Figure 5:**
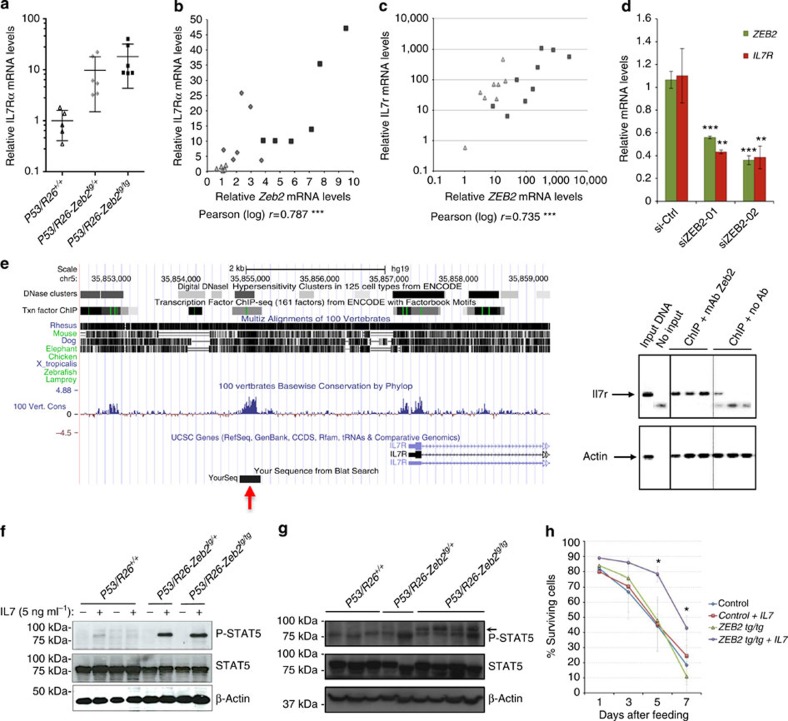
Strong positive correlation between *Zeb2* and *Il7r* mRNA levels associates with increased JAK/STAT signalling. (**a**) Quantitative Real-time (qRT)-PCR for *Il7r* in control *P53/R26*^*+/+*^ (*n*=5) and *Zeb2*-overexpressing *P53/R26-Zeb2*^*tg/+*^ (*n*=6) and *P53/R26-Zeb2*^*tg/tg*^ (*n*=6) thymic tumours. Mean±s.d. are indicated. (**b**) A correlation curve between *Zeb2* and *Il7r* mRNA levels. Pearson Correlation coefficient (*r*) was calculated. (**c**) qRT–PCR for *IL7R* and *ZEB2* in a cohort of human paediatric T-ALL patients; immature/ETP-ALL (*n*=9) and mature (*n*=8). A correlation curve between *ZEB2* and *IL7R* mRNA is shown. Pearson Correlation coefficient (*r*) was calculated. (**d**) qRT–PCR for *ZEB2* and *IL7R* after knockdown of *ZEB2* using two independent siRNAs in the immature human T-ALL cell line LOUCY. Results are presented as averages±s.d. of three electroporations/siRNA. Student’s *t*-test was used for statistical analysis. (**e**) Genomic alignment with multiple species showing that a highly conserved region in the *Il7r* promoter is located 2.5–3.0 kbp upstream of the transcription initiation start site (red arrow). Localization of the amplicon used for ChIP is depicted. Figure was made using UCSC genome bioinformatics software (http://genome.ucsc.edu/). ChIP was performed using a specific anti-ZEB2 monoclonal antibody in a *Zeb2*-overexpressing T-ALL cell line. PCR amplification was analysed via agarose gel electrophoresis. Each lane is an independent immunoprecipitation. (**f**) Western blot analysis for total STAT5 and P-STAT5 levels in control *P53/R26*^*+/+*^ and *Zeb2*-overexpressing *P53/R26-Zeb2*^*tg/+*^ and *P53/R26-Zeb2*^*tg/tg*^ T-ALL cell lines with and without recombinant murine IL-7 administration. (**g**) Western blot analysis for total STAT5 and P-STAT5 levels in control and *Zeb2*-overexpressing mouse thymic tumours. (**h**) The pro-survival effects of IL-7 administration on cell survival of control and *Zeb2*-overexpressing mouse T-ALL cell lines cultured under stressed conditions. Every line represents the average of two-cell lines/genotype. Student’s *t*-test was used for statistical analysis. Knockdown experiments in **d** were repeated three times using triplicate biological samples. Western blots (**f**,**g**) were performed twice and representative blots and graphs are shown. Images have been cropped for presentation. Full size images are presented in Supplementary Fig. 12. Survival experiments (**h**) were performed twice. *P<0.05, **P<0.01, ***P<0.001 (vs control).

Based on our microarray expression analysis and focusing on genes previously identified as recurrently mutated in immature/ETP-ALL^7^, we found a significant and *Zeb2* dosage-dependent increase in *Il7r* mRNA (Fig. 3a), which was confirmed by qRT–PCR analysis (a 8.5-fold increase in the *P53/R26-Zeb2*^*tg/+*^ and a 17-fold increase *P53/R26-Zeb2*^*tg/tg*^ tumours compared with p53/R26^+/+^ control tumours; Fig. 5a). As a result, a strong positive correlation (Pearson, *r*=0,787 was seen between *Zeb2* and *Il7r* mRNA expression levels in our mouse (Fig. 5b and Supplementary Fig. 10a) and human (Pearson, *r*=0.735) immature/ETP-ALL tumours (Fig. 5c and Supplementary Fig. 10b).

To functionally validate the observed correlative expression between *ZEB2* and *IL7R* in immature subtypes of human T-ALL, we performed short interfering RNA (siRNA)-mediated knockdown of *ZEB2* in the immature/ETP-ALL-like cell line LOUCY. *ZEB2* knockdown resulted in a significant downregulation of the *IL7R* mRNA levels (Fig. 5d), confirming the regulation of *IL7R* expression by ZEB2 in human leukaemic blasts. Moreover, the amplification of a phylogenetically conserved *Il7r* promoter element was enriched in a chromatin immunoprecipitation (ChIP) experiment using a ZEB2-specific monoclonal antibody, suggestive of direct binding of ZEB2 to this *Il7r* promoter element (Fig. 5e). However, *ZEB2* overexpression experiments in JURKAT, a mature T-ALL cell line that lacks the characteristic immature/ETP-ALL mRNA expression profile, did not yield any correlated expression with *IL7R* (Supplementary Fig. 10c), suggesting that an ETP-ALL-specific co-factor might be essential for the ZEB2-mediated effects on *IL7R* gene expression.

### ZEB2-induced *Il7r* upregulation promotes T-ALL cell survival

To functionally investigate the effects of increased *Il7r* levels and its downstream signalling by ZEB2, we initially tested the IL-7 effects on LOUCY cells with and without *ZEB2* knockdown. Also here a proportional decrease in *IL7R* levels could be observed (Supplementary Fig. 9b). However, as this cell line has lost its IL-7 responsiveness, we were unable to make use of this cell line to further study the functional links between *ZEB2* and *IL7R* signalling (Supplementary Fig. 9c–e).

To circumvent this issue, we utilized mouse T-ALL cell lines derived from two *P53/R26*^*+/+*^ control, one *P53/R26-Zeb2*^*tg/+*^ and four *P53/R26-Zeb2*^*tg/+*^ and *P53/R26-Zeb2*^*tg/tg*^ tumours. The *Zeb2*-overexpressing T-ALL cell lines have correlated increased *Il7r* mRNA and cell surface IL7R protein levels compared with the control cell lines (Supplementary Fig. 11a,b). In these cell lines, we monitored phosphorylated STAT5 (P-STAT5) levels, with and without recombinant IL-7 administration. Addition of the IL-7 ligand increased P-STAT5 levels in both *Zeb2*-overexpressing T-ALL cell lines, whereas no or minimal effect was observed in the two control cell lines (Fig. 5f). The latter might be associated with the differences in tumour immunophenotype between control *P53/R26*^*+/+*^ and *P53/R26-Zeb2*^*tg/tg*^ cells (Supplementary Fig. 11c). We conclude that the IL-7/STAT5 pathway is not mutated or constitutively activated in the *Zeb2*-overexpressing T-ALL cell lines, and remains responsive to ligand-induced activation. A similar increased level of P-STAT5 could be observed in primary tumour samples (Fig. 5g).

### Effects of interfering with ZEB2-mediated IL-7-JAK-STAT axis

**Figure 6:**
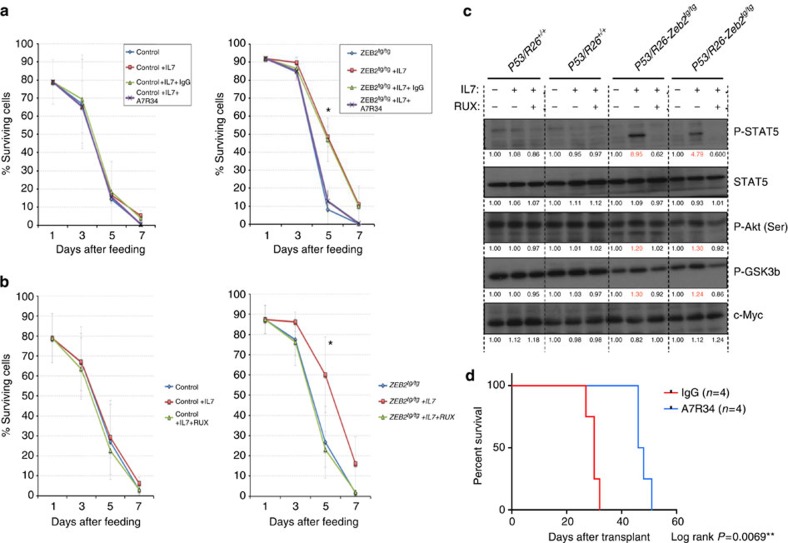
ZEB2 overexpression promotes T-ALL cell survival and can be prevented by blocking IL7R or JAK1/2 inhibition *in vitro* and *in vivo*. (**a**) The IL-7-dependent pro-survival effect of *Zeb2*-overexpressing cell lines can be prevented by co-administration of an IL7R-blocking antibody (A7R34), but not by the isotype control IgG. No effects are seen on the survival of the control cell lines. Student’s *t*-test was used for statistical analysis. (**b**) The IL-7-dependent pro-survival effect of *Zeb2*-overexpressing cell lines can be prevented by co-administration of 1 μg per ml Ruxolitinib phosphate (RUX), a JAK1/2 inhibitor. No effects are seen on the survival of the control cell lines. Student’s *t*-test was used for statistical analysis. (**c**) RUX effects were accompanied by the loss of the increased levels of phosphorylated P-STAT5 as well as phosphorylated AKT and GSK3β as determined by western blot analysis. Indicated quantifications represent the relative increase of signal compared with the signal observed without IL-7 and RUX administration. Student’s *t*-test was used for statistical analysis. (**d**) *In vivo* administration of the IL7R-blocking A7R34 antibody extended the lifespan of NOD/SCID mice transplanted with 2 × 10^6^ *P53/R26-Zeb2*^*tg/tg*^ cells. The experiments depicted in **a**–**c** were performed twice and representative graphs of western blots are shown. Images have been cropped for presentation. Full size images are presented in Supplementary Fig. 12. The experiment in **d** was performed once, using four mice per group. *P<0.05, **P<0.01 (vs control).

Based upon previous findings^26^,^27^, we hypothesized that *Zeb2*-overexpressing T-ALL cells (*P53/R26-Zeb2*^*tg/+*^ or *P53/R26-Zeb2*^*tg/tg*^) may be dependent upon IL-7 for optimal growth and/or survival. Indeed, we observed increased cell survival of the *P53/R26-Zeb2*^*tg/tg*^ T-ALL cell line in response to exogenous administration of IL-7, whereas only a minimal effect was observed in the control *P53/R26*^*+/+*^ cells (Fig. 5h). This *in vitro* pro-survival effect could be abolished through the use of a monoclonal IL7R-blocking antibody (A7R34; Fig. 6a) or the JAK1/2 inhibitor Ruxolitinib (RUX; Fig. 6b), and was accompanied by the loss of increased levels of P-STAT5 as well as phosphorylated AKT and GSK3β another pathway activated by IL-7 (Fig. 6c). No IL-7-dependent effects were seen on the proliferation rate of the cells (data not shown).

To test whether increased *Il7r* levels upon *Zeb2* upregulation are involved in the survival of the injected lymphoblast cells and enhanced leukaemia initiation, we transplanted *Zeb2*-overexpressing mouse cell lines (*P53/R26-Zeb2*^*tg/tg*^) with and without IL7R-blocking antibody (A7R34) administration. Extended survival of the transplanted NOD/SCID in the A7R34-treated animals was seen (Fig. 6d), indicating that enhanced *Il7r* levels and its downstream signalling is contributing to the increased leukaemia-initiating capacity of the *Zeb2*-overexpressing tumours.

## Discussion

Whole-genome expression profiling of T-ALL patients has defined immature/ETP-ALLs as a distinct entity^4^,^5^,^6^, characterized by a poor response to current treatment regimens. A recent whole-genome sequencing study suggested that this immature T-ALL subgroup is genetically heterogenous, and although multiple recurrent genomic lesions have recently been identified^7^, the molecular drivers and therapeutic targets remain elusive. Here we provide compelling evidence for an oncogenic driver role for ZEB2 through gain-of-function mechanisms in immature/ETP-ALL development.

First, we observed increased *ZEB2* levels in the paediatric and adult immature/ETP-ALL subclass and identified a rare but recurrent t(2;14)(q22;q32) translocation in a few of these typical ETP-ALL cases. This novel translocation, results in juxtaposition of the promotor and proximal portion of the *BCL11B* locus to the ZEB2 locus, a finding reminiscent of previously described *BCL11B*-driven T-ALL oncogene activation^12^,^13^. These data are strongly indicative for an oncogenic driver role for ZEB2 in T-ALL, and is consistent with previously reported retroviral mutagenesis screens^28^,^29^,^30^ that have suggested *Zeb2* involvement in leukaemogenesis. Whether or not loss/alternative functions of BCL11B are involved in leukaemia initiation/progression specifically in these patients remains unknown. Importantly, other mechanisms are involved in ETP-ALL patients leading to *ZEB2* upregulated expression levels besides these rare recurrent translocations. One potential mechanism may be related to altered expression of miR200c, which have previously been shown to negatively regulate *Zeb* family member expression^11^,^31^ and to be altered in cancer settings. Exactly how miR200c levels are downregulated to begin with remains to be determined but may be related to changes in the promoter methylation status as has been previously shown in other tumour types^32^,^33^.

Using a *ROSA26*-based overexpression of *Zeb2* in the endothelium and throughout the entire haematopoietic system induces formation of precursor T-cell lymphoblastic leukaemia. Using the T-cell-restricted CD4-Cre line to overexpress *Zeb2* recapitulated the spontaneous thymoma formation and strongly suggests a cell autonomous role of *Zeb2*, however, given the paracrine effects associated with *Zeb2* deletion in the central nervous system^34^, we cannot exclude environmental involvement in the observed T-cell lymphoma formation phenotype. Intercrossing onto a p53-deficient background drastically accelerated tumour formation and shifted the tumour spectrum towards an immature precursor T-cell lymphoblastic leukaemia, with an expression profile similar to ETP-ALL patients. Mouse tumours showed prototypical activating mutations affecting *Notch1* and loss of the tumour-suppressor genes *Pten* and *Ikzf1*, supporting the fact that the p53 null *Zeb2* transgenic mouse model closely recapitulates human T-cell leukaemia^35^,^36^,^37^, and is in keeping with the higher occurrence of *IKZF1* mutations in ETP-ALLs^7^.

In line with a presumed ETP-ALL phenotype for the mouse *Zeb2*-driven leukaemias, we also observed increased LSC properties. These enhanced LSC characteristics associated with *Zeb2* overexpression are in line with previous observations that expression of the ZEB family members is correlated with poor prognosis of solid tumours^8^,^38^, putatively in part through the acquisition of enhanced cancer stemness programmes^10^,^11^.

Mechanistically, we demonstrate that ZEB2 leads to upregulated *IL7R* expression in immature/ETP-ALL cells. A strong positive correlation was observed between *Zeb2* and *Il7r* mRNA levels in our mouse model and in the human LOUCY cell line, which exhibits an ETP-ALL-like phenotype. IL-7 signalling is of key importance in normal thymocyte maturation and differentiation and constitutive activation of IL7R-driven signalling has been shown to lead to T-ALL oncogenesis. In approximately 10% of T-ALLs, including ETP-ALLs^7^,^39^,^40^,^41^, activating mutations in the *IL7R* gene are present representing an interesting drugable target for novel treatment using IL7R-blocking antibodies or more novel compounds for the inhibition of downstream JAK/STAT5 signalling^27^, like RUX. Previous studies have demonstrated that use of blocking IL-7 antibodies or use of IL-7-deficient mice could dramatically decrease human T-ALL formation in xenotransplantation settings using immunocompromised mice^27^,^42^. Here we could see a clear IL-7-dependent survival effect of the derived *Zeb2*-overexpressing mouse T-ALL cell lines *in vitro* and an effect on their ability to initiate secondary tumours after transplantation. Whether the described ZEB2-IL7R axis is also important in human ETP-ALL disease progression remains to be tested.

In conclusion, we have found that sustained overexpression of *Zeb2* acts as a leukaemic driver for immature T-ALLs with increased leukaemic stem cell properties. ZEB2-mediated upregulation of *Il7r* expression and activation of the JAK/STAT pathway represents a possible therapeutic target for this aggressive and chemoresistant subtype of human T-ALL.

## Methods

### Patients

Conventional karyotyping was performed in bone marrow samples at T-ALL diagnosis. Patients TL88 and Case #2 were both adults. Karyotype was 46,XY,t(2;14)(q22;q32)[20] in both cases. Leukaemic cells immunophenotype was as follows for TL88: CD2^+^, CD5^weak^, CD7^+^, CD3ic^+^, CD4^−^, CD8^−^, CD34^+^, CD13^+^, CD33^−^, CD10^−^, B-cell marker negative; and Case #2: CD2^+^, CD5^weak^, CD7^+^, CD3ic^+^, CD34^+^, CD33^−^, CD13^+^, B-cell markers negative. Patients provided informed consent to use leftover material for research purposes according to the Helsinki Declaration. This study was approved by the Institutional Review Board of the Institut Universitaire d’Hématologie, Paris, France.

Two other T-ALL patients with a similar translocation were identified via conventional karyotype analysis in a an independent cohort: Case #3 (43-year-old) 46,XY,t(2;14)(q23;q32)[19]/ 46,idem,del(6)(q?13q?15)[17]/46,XY[2] and Case #4 (6-year-old) 46,XY,+Y,t(2;14)(q23;q32),del(7)(q3?6),del(9)(q22),-12,der(19)t(12;19)(q13;?p/q)[7]. Also for these patients, local ethical committee approval was obtained by the treatment centre and informed consent was given according to the Helsinki Declaration.

### Animal experimentation and handling

All experiments were performed according to the regulations and guidelines of the Ethics Committee for care and use of laboratory animals of Ghent University. The mouse (*Mus musculus*) cohorts used in these experiments were sibling littermates and were maintained on a mixed CD1 outbred genetic background and randomly grouped into control and experimental groups based upon their genotypes. Both male and female mice were included in the various analyses and ranged in age from 3 to 60 weeks.

For the transplantation experiments, primary thymic tumours were dissected under aseptic conditions. Cell concentrations were measured using Burker cell counter chamber. A dilution series was prepared in sterile PBS and the indicated cell numbers were intravenously injected in 6- to 10-week-old NOD/SCID recipient mice (Charles River). A similar approach was used for the transplantation of the derived murine cell lines. Three or four primary tumours per genotype and at least two independent cell lines/genotype were used.

For the transplantation experiments in combination with IL7R-blocking antibody, 2 × 10^6^ *P53/R26-Zeb2*^*tg/tg*^ cells in sterile PBS were intravenously injected in eight NOD/SCID recipient mice (Charles River). After injection, the mice were randomly divided in two groups, and treatment was started. For 3 weeks after transplantation, mice were intraperitoneally injected every second day with 450 μg IL7R-blocking antibody (A7R34; BioXCell) or 450 μg isotype control antibody (2A3; BioXCell) in case of the control group.

5 × 10^6^ luciferase-labelled LOUCY cells in sterile PBS were injected in 6-week-old (NSG KO mice) via the tail vein (*n*=3 per cell line). At regular time points, the bioluminescence was measured using the IVIS Lumina II imaging system (Perkin Elmer). Before imaging, the mice were injected intraperitoneally with 200 μl of a 15 mg ml^−1^ firefly D-Luciferin potassium salt solution and anaesthetized by inhalation of 5% isoflurane. The mice were imaged 10 min after Luciferin injection. Transplantation experiments using leukaemic cell lines were not repeated in order to reduce the number of laboratory animals used on request of the ethical committee of Ghent University.

### Mouse pathology

A complete necropsy was performed on each mouse included in Supplementary Table 1. Pathological and histological examination were performed in a blinded manner. Tissue samples were formalin-fixed, paraffin-embedded, sectioned and stained with haematoxylin and eosin for histopathological examination. Additional immunohistochemical (IHC) analysis was performed when necessary.

Samples of gross lesions, brain, skin, salivary glands, trachea, oesophagus, thyroids, lungs, heart, spleen, liver, kidneys, pancreas, gastrointestinal tract, thymus, lymph nodes (cervical, pancreatic and mesenteric), urogenital tract, adrenals and lumbar vertebrae (bone marrow) were formalin-fixed, paraffin-embedded, sectioned at 4 μm and stained with haematoxylin and eosin for histopathological examination. Additional IHC analyses were also performed in order to better characterize the origin of the neoplastic lesions encountered. Details concerning the panel of IHC stains applied are reported in Supplementary Table 4. The different categories of lymphoid and vascular tumours were defined according to the diagnostic criteria proposed by Morse *et al*.^43^ and Rehg and Ward^44^, respectively. Detailed definition of tumour categories is given below:

*Pre-T LBL: thymic*: Pre-T LBL characterized by primary thymic involvement with different degree of local invasion (for example, mediastinum, lungs and heart) but without distant spread to extrathoracic organs/tissues. Immunophenotype (as determined by means of immunohistochemistry): CD45/CLA^+^; CD3^+^; cKit ^+*^; CD45/B220^−^; CD20^−^; IBA-1^−^.

*Pre-T LBL/L: generalized/systemic*: Pre-T LBL//leukaemia characterized by primary thymic involvement with different degree of local invasion (for example, mediastinum, lungs and heart) and distant spread to/involvement of extrathoracic organs/tissues including spleen, superficial and internal lymph nodes, bone marrow, liver and kidneys. Increased white blood cell count with more than 20% of immature and atypical lymphoid form was also documented in peripheral blood of some cases. Immunophenotype (as determined by means of immunohistochemistry): CD45/CLA^+^; CD3^+^; cKit^+*^; CD45/B220; CD20^−^; IBA-1^−^.

*Uncharacterized (non-thymic) T L: multicentric*: not otherwise characterized T-cell lymphoma with primary involvement of superficial and internal lymph nodes, gut associated lymphoid tissue (GALT) and spleen. The neoplasm displays different degree of local invasion and massive spread to liver, kidneys, lungs, pancreas, gonads and central nervous system. Thymus and bone marrow are distinctively spared. Immunophenotype (as determined by means of immunohistochemistry): CD45/CLA^+^; CD3^+^; CD45/B220^−^; IBA-1^−^.

*ML*: myeloid leukaemia with neutrophilic differentiation arising from bone marrow and spleen and with different degree of hepatic, renal and nodal involvement. Leukaemic population in affected organs is mainly composed of myeloblasts and immature granulocytic forms (promyelocytes and myelocytes). Increased white blood cell count with prevalence of myeloblasts and immature neutrophilic forms is also evident in the peripheral blood.

*SMZL*: sSplenic marginal zone lymphoma. Immunophenotype (as determined by means of immunohistochemistry): CD45/CLA^+^; CD3^−^; CD45/B220^+^; IBA-1^−^.

*HMA*: haemangioma, solitary begnign neoplasm of vascular endothelial origin (CD31^+^ and Lyve1^−^ immunohistochemistry) arising from mediastinum or retroperitoneal space.

*HSA solitary:* haemangiosarcoma, solitary malignant neoplasm of vascular endothelial origin (CD31^+^ and Lyve1^−^ immunohistochemistry) arising from retroperitoneal space or subacutis/muscular fascia of the trunk.

*HSA multicentric*: multicentric HSAs, multiple malignant neoplasms of vascular endothelial origin (CD31^+^ and Lyve1^−^ immunohistochemistry) arising simultaneously from two or more of the following locations: subacutis/muscular fascia of the trunk, heart, liver, spleen, gonads.

*HSA multicentric with metastases*: —multicentric HSAs, multiple malignant neoplasms of vascular endothelial origin (CD31^+^ and Lyve1^−^ immunohistochemistry) arising simultaneously from heart and subacutis/muscular fascia of the trunk and metastasizing to the lungs.

*PA*: solitary pulmonary adenoma.

*IPA:* solitary intestinal polypoid adenoma.

*NGCO*: solitary nongestational choriocarcinoma of the ovary.

*Variable degree of cKit expression with occasional negative cases. According to Rehg *et al*.^45^, neoplasms with consistent cKit expression can be classified as early Pre-T LL/L (presumably originating from CD4, CD8 double negative (DN) thymocytes), whereas neoplasms with minimal or negative cKit expression should be considered as late Pre-T LL/L (presumably originating from DN thymocytes).

For IHC details see Supplementary Table 4.

### Flow cytometry

Cells were analysed by LSRII (BD Biosciences) and FACSDiva or FlowJo software (BD Biosciences). Cell debris and cell aggregates were gated out, dead cells were discarded using Propidium Iodide or the fixable Viability Dye eFluro506 (eBioscience). Antibodies used for flow cytometry are listed in Supplementary Table 5. Intracellular stainings were done using BD Cytofix/Cytoperm kit (BD Bioscience) according to the manufacturer guidelines.

### Cell culture

Primary mouse thymic lymphoma cell lines were generated according to the previously published protocols^46^. In short, thymus tumours were dissected under aseptic conditions and plated in RPMI medium supplemented with 10% heat-inactivated fetal bovine serum (Sigma), penicillin (100 U ml^−1^)-streptomycin (100 μg ml^−1^), 2 mM L-glutamine (Gibco), 0.05 mM 2-mercaptophenol and where indicated 5 ng ml^−1^ recombinant IL-7 (Peprotech) and incubated at 37 °C with 5% CO_2_ and 95% humidity. After 4–5 passages, stable cell cultures were obtained and freezings were made for further use.

For IL-7 effects on cell survival and proliferation, 3 × 10^6^ primary T-ALL cells were labelled with 5 ng ml^−1^ Cell Proliferation Dye eFluor670 (eBioscience) for 10 min at 37 °C, washed three times with cold medium and seeded at a concentration of 1 × 10^6^ cell per ml in medium with or without 5 ng ml^−1^ IL-7 and incubated at 37 °C, 5% CO_2_ and 95% humidity. Every second day, the percentage of surviving cells was quantified using the fixable Viability Dye eFluro506 (eBioscience) and flow cytometry. Where indicated JAK1/2 signalling was inhibited by administration of RUX phosphate (INCB018424; InvivoGen). A stock solution of 20 mM RUX was prepared in dimethylsulphoxide and diluted in growth medium to a working dilution of 1 μM. Where indicated, IL7R signalling was blocked by administration of 100 μg ml^−1^ A7R34-blocking antibody (BioXCell). As a control, we used 100 μg ml^−1^ isotype control antibody 2A3 (BioXCell). These *in vitro* cell survival experiments were performed on at least two independent primary cell lines/genotype and repeated twice/experiment.

The LOUCY cell line (DSMZ, Germany) was cultured in RPMI-1640 media supplemented with 10% fetal bovine serum, 100 U ml^−1^ penicillin G, 100 μg ml^−1^ streptomycin, 100 μg ml^−1^ kanamycin and 2 mM L-glutamine at 37 °C in a humidified atmosphere under 5% CO_2_. For siRNA-mediated knockdown experiments, we performed electroporation in triplicate of 400 nM *ZEB2*-specific siRNAs (siZEB2-01: ON-TARGETplus Human ZEB2 siRNA J-006914-07, siZEB2-02: Thermo Scientific; Silencer Select Pre-designed Human ZEB2 siRNA s19034, Life Technologies) or 400 nM of scrambled siRNAs (si-Ctrl: siGENOME Non-targeting siRNA pool #2, Thermo Scientific) as control. Also electroporation without adding siRNAs was performed as control (no siRNA). For the electroporation, an exponential decay pulse (300 V, 1,000 μF; Genepulser MxCell, Bio-Rad) was used. After 72 h, RNA was isolated and evaluated by qRT–PCR. Results were presented as mRNA expression relative to control electroporation results. siRNA knockdown results where siRNA did not result in a proper *Zeb2* knockdown were excluded.

Luciferase-positive LOUCY cells were generated by infecting the LOUCY cell line with a PWPI-LUC lentivirus. Non-concentrated virus was produced in HEK293TN cells using JetPEI polyplus reagent with pMD2.G (envelope plasmid), psPAX2 (packaging plasmid) and pWPI-LUC (target plasmid) in 0.1/0.9/1 ratios. Luciferase activity was verified *in vitro* using the Dual-Luciferase Reporter Assay kit (Promega). Stable knockdown for ZEB2 in LOUCY were generated as described previously^47^. Lentiviral particles were generated using pLV-TH shZEB2(SEC8) or the pLV-TH empty vectors. Transduction efficiencies were determined by measuring enhanced green fluorescent protein (EGFP) expression using flow cytometry. Subsequently, the cells were sorted using a FACSAria II machine (BD Biosciences) to obtain cell populations with more than 90% EGFP-positive cells. All original T-ALL cell lines were obtained from the DSZM (http://www.dsmz.de/) at the start of this study and tested and found to be free of mycoplasma contamination.

### Real-time quantitative PCRs

Total RNA was isolated using RNeasy Plus Mini Kit (Qiagen). cDNA was synthesized using the First Strand cDNA Synthesis Kit (Roche) with oligo(dT) primer, starting from equal amounts of RNA. qRT–PCRs were performed using the LightCycler 480 SYBR Green I Master (Roche), monitored on a LichtCycler 480 system (Roche) and analysed using qBase software from Biogazelle. Gene expression was standardized against housekeeping genes β-actin, glyceraldehyde-3-phosphate dehydrogenase (Gapdh), RPL13 and TBP. All primers used are listed in Supplementary Table 6. In the qRT–PCR analysis of human T-ALL patients, results of two mature T-ALL and one immature T-ALL cases were excluded because of high variability within the housekeeper genes or between technical replicates, most probably due to low RNA quality.

### Gene expression profiling

For human T-ALL patients, expression data of previously published gene expression profiling arrays of two independent cohorts of T-ALL patients (one paediatric and one adult cohort) was used. The first cohort consisted of bone marrow lymphoblast samples from 64 paediatric T-ALL patients (15 immature, 25 *TAL/LMO*, 17 *TLX1/TLX3* and 7 *HOXA*), which were collected with informed consent according to the declaration of Helsinki from Saint-Louis Hospital (Paris, France). Transcriptome profiling was performed on GeneChip Human Genome U133 2.0 Plus arrays (Affymetrix) according to the standardized procedures at the IGBMC (www.igbmc.fr, Strasbourg, France) and microarray data files are available from ArrayExpress under accession no. E-MTAB-604 (ref. 16). The second cohort consisted of 57 cryopreserved lymphoblast samples, which were provided by the Eastern Cooperative Oncology Group. All samples were collected in clinical trials E2993 and C10403. Gene expression profiling was performed using the HumanHT-12 v4 Expression BeadChip (Illumina) and microarray data files are available from the Gene Expression Omnibus accession nr GSE33469 (ref. 17). Expression data of the mi-R141/200c cluster were obtained via a quantitative PCR-based microRNA profilering study on 15 immature and 25 mature primary T-ALLs^48^.

From the mouse leukaemia, RNA was isolated using the miRNeasy mini kit with DNA digestion on-column (Qiagen). RNA concentration was measured on the NanoDrop 1000 Spectrophotometer followed by RNA quality assessment by use of the Experion Automated Electrophoresis System according to the manufacturer’s instructions (Bio-Rad). Next, RNA samples were profiled for gene expression analysis on SurePrint G3 Mouse 8 × 60 K Microarrays according to the manufacturer’s instructions (Agilent Technologies). Normalization of gene expression data was done by quantile normalization using R. Differential gene expression analysis was performed using fold change analysis and *P*-value calculation based on unpaired *t*-test. Microarray data files are available from the Gene Expression Omnibus accession number GSE62653. Gene set enrichment analysis was executed against annotated gene set of a 500-probe set enriched in human immature/ETP-ALL. Gene profiling was originally performed on three *P53/R26*^*+/+*^ vs three *P53/R26-Zeb2*^*tg/tg*^ thymic tumours, however, one of the control *P53/R26*^*+/+*^ had spontaneously high levels of Zeb2 that occurred and was therefore excluded from the control group. In all aspects, the phenotype of this one control mouse tumour (decreased tumour latency, *cKit* expression, high *Lyl1*, *Mef2c*, *Hhex* and *Il7r* expression) resembled the phenotype of the *Zeb2*-overexpressing tumours, indicating that similarly high levels of *Zeb2* can be spontaneously observed in human and mouse immature T-cell lymphoblastic leukaemias (Supplementary Fig. 8). This sample was also removed from the qRT–PCR analysis depicted in Figs 4c and 5a and Supplementary Fig. 6.

### Chromatin immunoprecipitation

*P53/ROSA26-Zeb2*^*tg/tg*^ primary mouse T-ALL cells were cross-linked in 1% formaldehyde for 10 min at room temperature and stopped with glycine (final concentration 0.125 M). Cells were lysed in the presence of protease inhibitors and DNA was sonicated. For immunoprecipitation, 100 μg DNA was used together with 3 μl of anti-ZEB2 monoclonal Ab^49^. Complexes were precipitated with protein A and G Sepharose beads (GE Healthcare). Formaldehyde cross-links were reversed by overnight incubation at 65 °C and DNA was purified using QIAquick PCR Purification Kit (Qiagen). Primers used are given in Supplementary Table 6, including localization of the respective amplicon on the mouse *Il7r* promotor (Supplementary Fig. 10c). Figure was generated using USCS genome bioinformatics software (http://genome.uscs.edu/).

### Western blotting

Protein lysates of IL-7 non-induced and induced (10 ng ml^−1^, 30 min) T-ALL cell lines were collected in Laemmli buffer (62.5 mM Tris–HCl, pH 6.8; 10% glycerol; 2% SDS and 5% β-mercaptoethanol) containing phosphatase and protease inhibitors (Roche) and sonicated before analysis. Total protein concentration was determined using DC Protein Assay (Bio-Rad). 40 μg per sample was subjected to SDS–polyacrylamide gel electrophoresis, transferred onto a membrane (Millipore) and used for detection. Primary antibodies used for detection: rabbit polyclonal anti-STAT5 (1:1,000; #9363, Cell Signaling), rabbit polyclonal P-STAT5(Tyr694) (1:1,000; #9351, Cell Signaling), mouse monoclonal P-AKT(Ser473) (1:1,000; #4051S, Cell Signaling), rabbit monoclonal P-GSK3β (Ser9) (1:1,000; #9323, Cell Signaling), rabbit polyclonal cMyc (1:1,000; #9402S, Cell Signaling) and mouse anti-actin (1:10,000; clone C4, Molecular probes, Invitrogen). Anti-mouse and anti-rabbit IgG-HRP secondary antibodies (1:8,000; Sigma-Aldrich) and the ECL detection kit (GE Healthcare) were used for detection.

Western blot quantifications were done using Fiji software. To rule out loading differences, quantifications were normalized using signal detected for loading control β-actin. Western blot analysis was performed on at least two independent primary cell lines/genotype and repeated twice/experiment.

Images have been cropped for presentation. Full size images are presented in Supplementary Fig. 12.

### Mutation analysis on mouse tumour samples

Mutation analysis was performed on *Notch1* (exons 26, 27 and 34), *Fbxw7* (exons 8, 9 and 11) and *Pten* (exons 5 and 7) as previously described^50^ and using primers shown in Supplementary Table 6. Briefly, the PCR amplification mix consisted of 20 ng of DNA, 1 × KapaTaq reaction buffer (KapaBiosystems), 1 U KapaTaq DNA polymerase, 0.2 mM dNTP, 2.5 μM MgCl2, 0.2 mM forward and reverse primer in a 25 μl of PCR reaction. Next, following PCR protocol was performed: 10 min at 95 °C, 40 cycles (15 s at 96 °C, 1 min at 57 °C and 1 min at 72 °C) and 10 at min 72 °C. Finally, PCR products were enzymatically purified followed by primer extension sequencing and electrophoresis using ABI3730xl (Applied Biosystems).

### Fluorescence *in situ* hybridization

Bacterial artificial chromosome (BAC) probes were obtained from the Roswell Park Cancer Institute (https://www.roswellpark.edu/shared-resources/genomics/services-and-fees/genomic-clone-distribution. For the *BCL11B* locus at 14q32.2, flanking probes RP11-68I8, RP11-889B13 or RP11-145P8, RP11-15E14 were used. To map the breakpoint locus on chromosome 2q22, sets of flanking probes were used in a chromosome walk on metaphase spreads from case TL88, leading to map the ZEB2 locus. A second translocation *BCL11B-ZEB2* was then demonstrated in the second patient (Case #2) using hybridization of combination of probes RP11-68I8, RP11-889B13, RP11-224H3 and RP11-67J2.

### Statistics and general methods of data analysis

Kaplan–Meier survival curves for the various mouse cohorts used in this study were generated with GraphPad/Prism6 software and Log-rank (Mantel–Cox) test was performed for statistical analysis. Groups of at least ten mice per cohort were randomly used per genotype in order to obtain statistical significance.

Data analysis for differential expression analysis via real-time PCR was performed using the qBase+ (Biogazelle) or GraphPad/Prism6 software and standardized against at least three housekeeping genes. Data were presented as average±s.d. and indicated in the figure or figure legend. Comparison between two data groups was done by two-sided Student’s *t*-test. One-way ANOVA test was used for statistical analysis between three or more data groups. The Mann–Whitney *U*-test was used to analyse differences in ZEB2 expression between genetic subtypes of human T-ALL patients (microarray profile). The data sets used in the various statistical analyses showed normal distribution around the mean and similar variance between the groups that were being statistically compared. Unless otherwise stated, all experiments were performed using at least three biological replicates and experiments. *P<0.05, **P<0.01, ***P<0.001 (vs control).

## Additional information

**How to cite this article**: Goossens, S. *et al*. ZEB2 drives immature T-cell lymphoblastic leukaemia development via enhanced tumour-initiating potential and IL-7 receptor signalling. *Nat. Commun.* 6:5794 doi: 10.1038/ncomms6794 (2015).

## Supplementary Material

Supplementary Information Supplementary Figures 1-12, Supplementary Tables 1-6 and Supplementary References.
