# Supplementary material for: ZEB2 drives immature T-cell lymphoblastic leukaemia development via enhanced tumour-initiating potential and IL-7 receptor signalling
Source: Nat Commun. 2015 Jan 7;6:5794. doi: 10.1038/ncomms6794 (PMC4354161; doi:10.1038/ncomms6794)
Supplement: Supplementary Information — Supplementary Figures 1-12, Supplementary Tables 1-6 and Supplementary References. [file ncomms6794-s1.pdf]

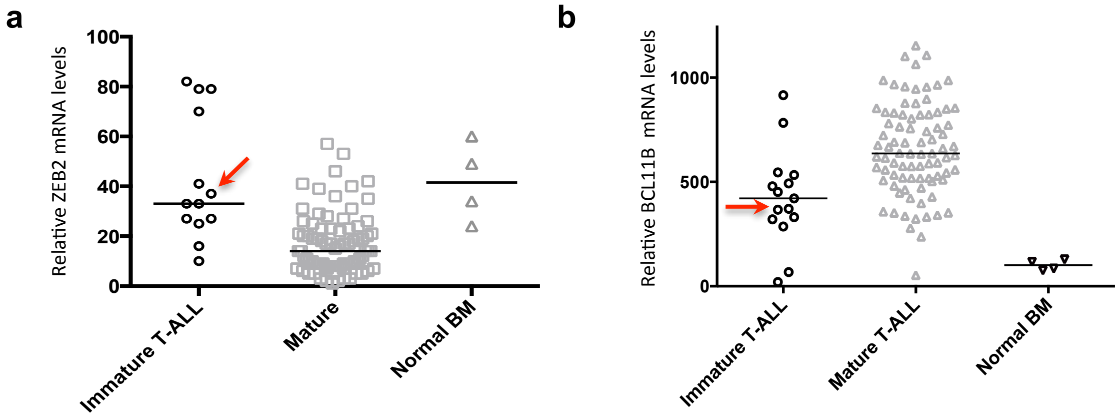

Supplementary Figure S1. *ZEB2* and *BCL2* levels in t(2;14)(q22;q32) patient TL88

(a) *ZEB2* expression profile of a previously published cohort of 92 T-ALL patients[1]. The red arrow indicates the *ZEB2* mRNA expression level for patient TL88 with t(2;14)(q22;q32). T-ALL patients are, grouped into immature and mature subclasses based on an unbiased hierarchical clustering of their gene expression profile. Mean expression is indicated. **(b)** *BCL11B* expression profile with a red arrow indicating the expression level for patient TL88 with t(2;14)(q22;q32). Mean expression is indicated.

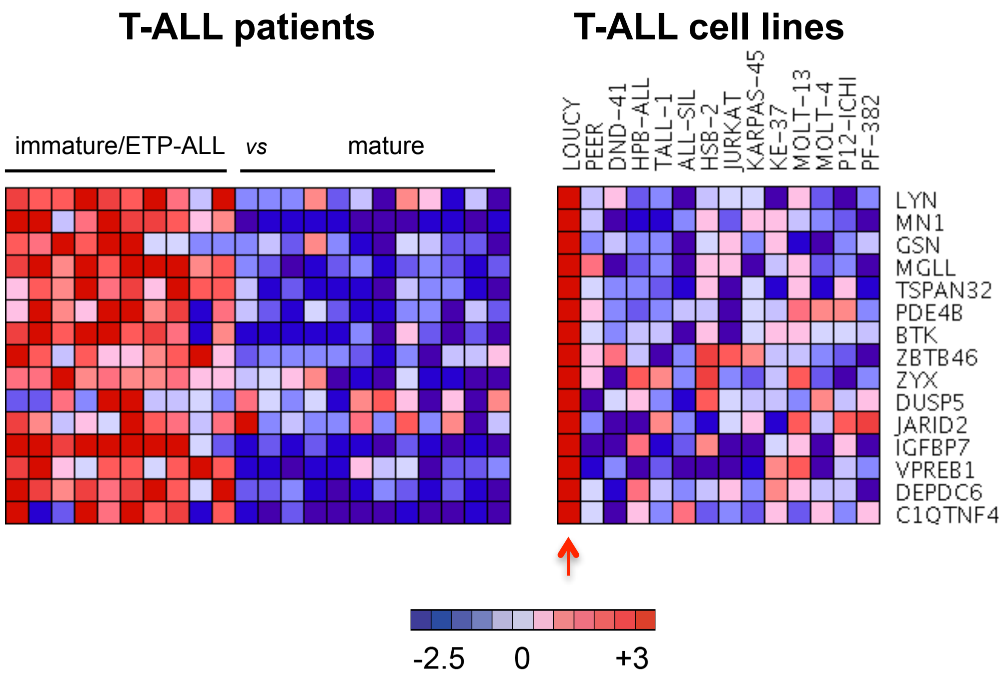

Supplementary Figure S2. Characterization of available human T-ALL cell lines via micro-array profiling

Expression profile of the top 15 genes differentially expressed in a published cohort of adult T-ALL patients (Left panel)[2] and compared to their expression in the available human T-ALL cell lines (Right panel). The only T-ALL cell line that shows an immature/ETP-ALL expression profile is LOUCY, indicated by a red arrow.

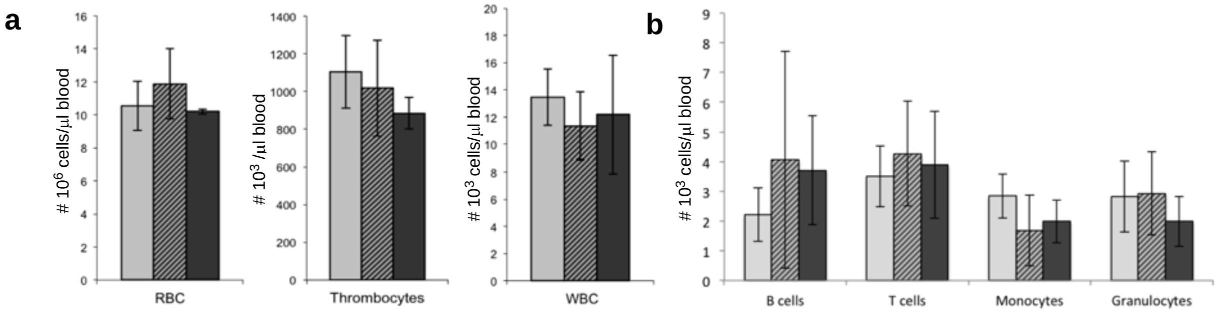

Supplementary Figure 3. Peripheral blood analysis of the *Tie2cre, R26-Zeb2$^{tg/tg}$* mice (a) Peripheral blood analysis of *Zeb2* overexpressing mice compared to control littermates (n=4/ genotype) using 5-differential automated blood analyzer (Hemavet, Drew Scientific), **(b)** in combination with flow cytometry.

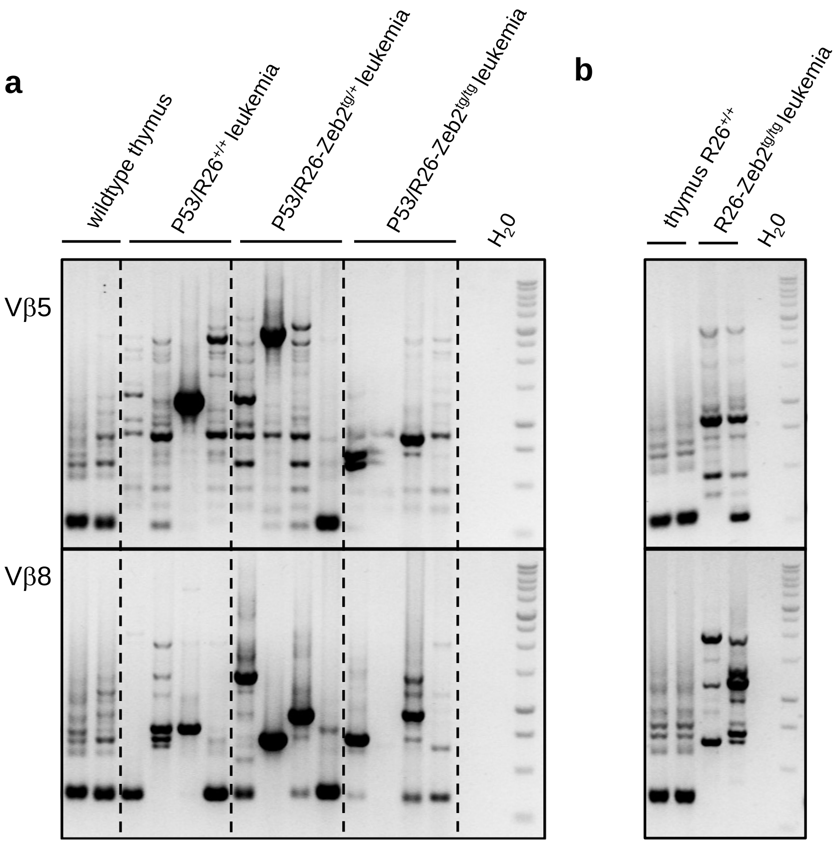

Supplementary Figure 4. TCR rearrangement analysis showing mono/oligoclonal origin of Pre-T LBL

(a) PCR analysis of genomic DNA isolated from control thymus (WT) and *P53/R26*$^{+/+}$, *Zeb2* overexpressing *P53/R26-Zeb2*$^{tg/+}$ and *P53/R26-Zeb2*$^{tg/tg}$ thymic tumors demonstrating the mono/oligoclonal expansion of transformed thymocytes examined by Vβ5 an Vβ8 TCR rearrangement products. **(b)** PCR analysis of genomic DNA isolated from control thymus (WT) and *R26-Zeb2*$^{tg/tg}$ thymic tumors demonstrating the mono/oligoclonal expansion of transformed thymocytes examined by Vβ5 and Vβ8 TCR rearrangement products.

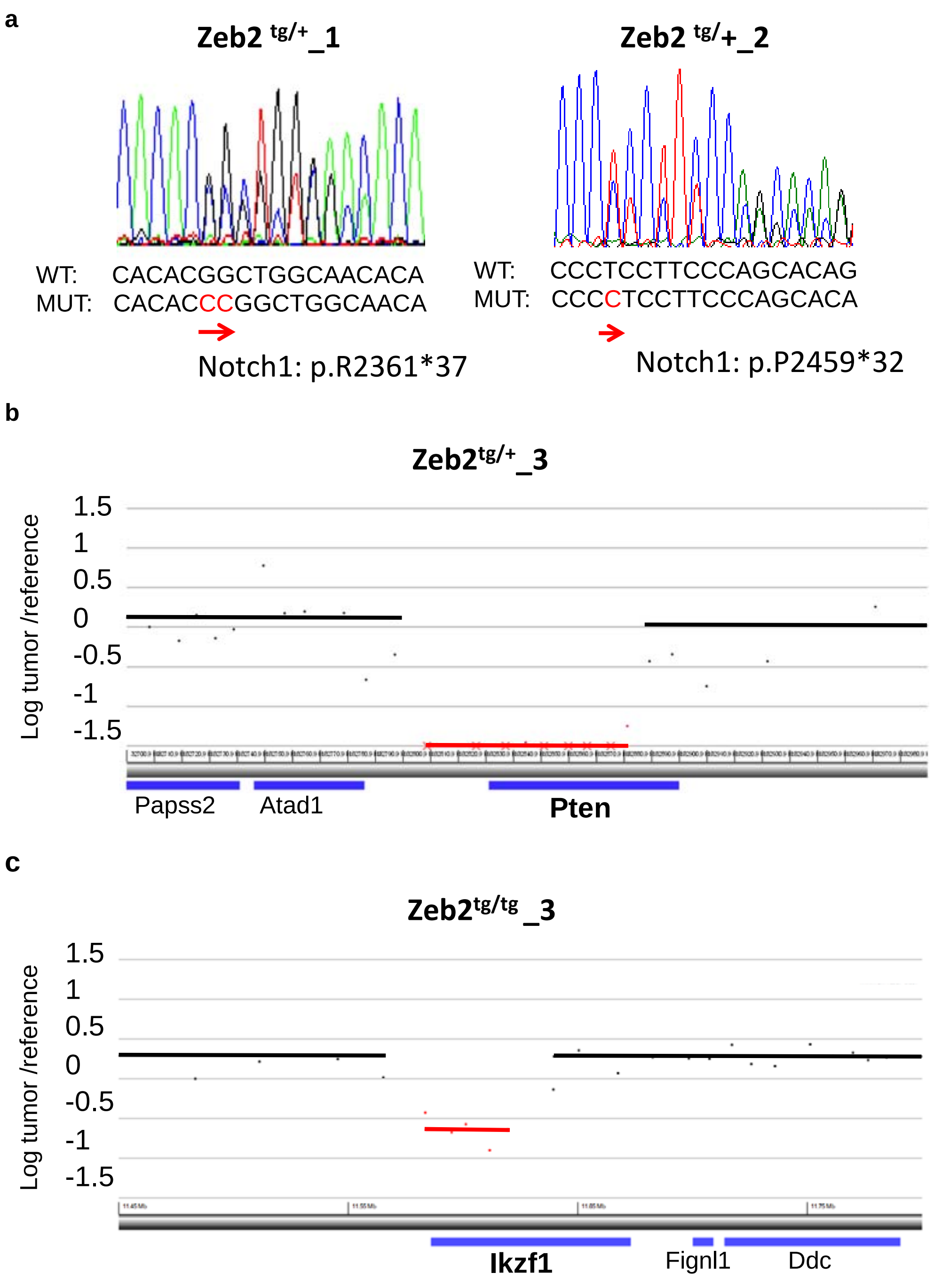

Supplementary Figure 5. Mutation analysis of control *P53/R26*$^{+/+}$ and *Zeb2* overexpressing *P53/R26-Zeb2*$^{tg/+}$ and *P53/R26-Zeb2* $^{tg/tg}$ thymic tumors

(a) Sequence of two *Notch1* gain-of-function point mutations found in *Zeb2* overexpressing tumors (P53/R26-Zeb2$^{tg/+}$) **(b)** *Pten* loss-of-function mutation and **(c)** Recurrent *Ikzf1* loss-of-function mutation found in *Zeb2* overexpressing (*P53/R26-Zeb2*$^{tg/+}$ or *P53/R26-Zeb2*$^{tg/tg}$) thymic tumors.

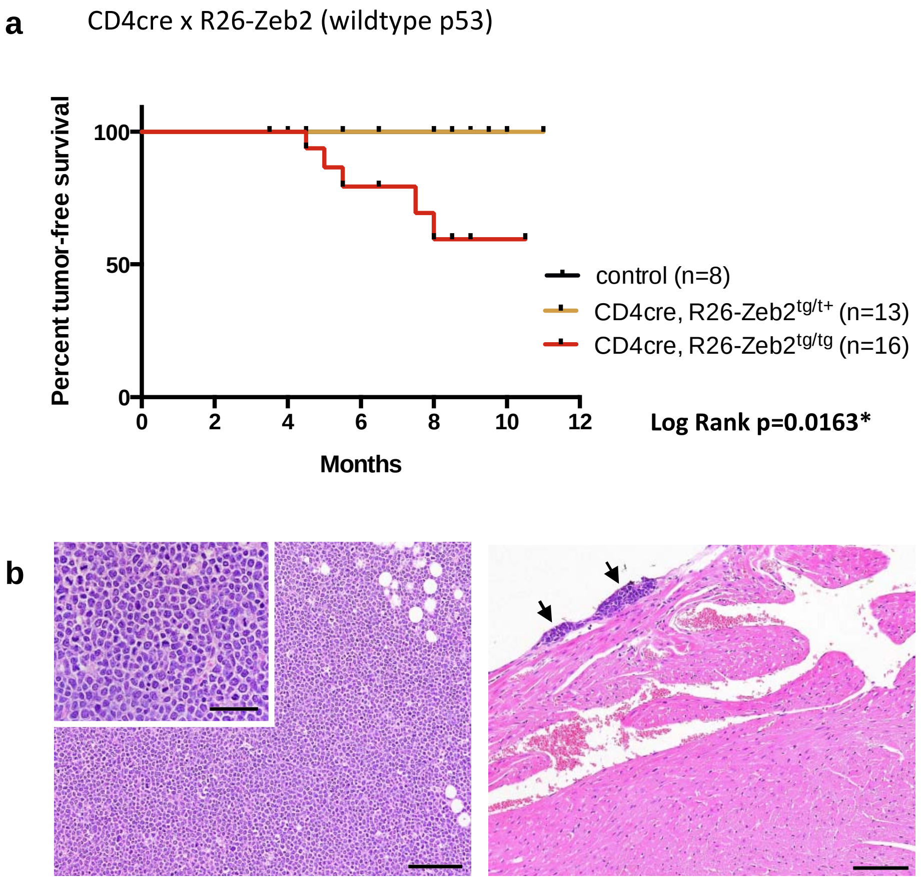

Supplementary Figure 6. Overexpression of *Zeb2* using a T-cell restricted Cre line, CD4-cre

(a) Kaplan-Meier survival curve of *CD4-Cre, R26-Zeb2$^{tg/tg}$* mice (n=16). Mantel-Cox test was used for statistical analysis. **(B)** Pathological examination diagnosed these mice with T-cell lymphoblastic leukemia. Left panel; Cranial mediastinum, dense neoplastic infiltrates characterized by medium to large-sized atypical lymphoid cells with numerous mitotic figures (inset). H&E stain, scale bar = 100 µm (main panel) and 50 µm (inset). Right panel; dissemination to the heart, multifocal infiltrates of medium to large-sized atypical lymphoid cells expanding the epicardium of the right ventricular free wall (arrows). H&E stain, scale bar ,100 µm.

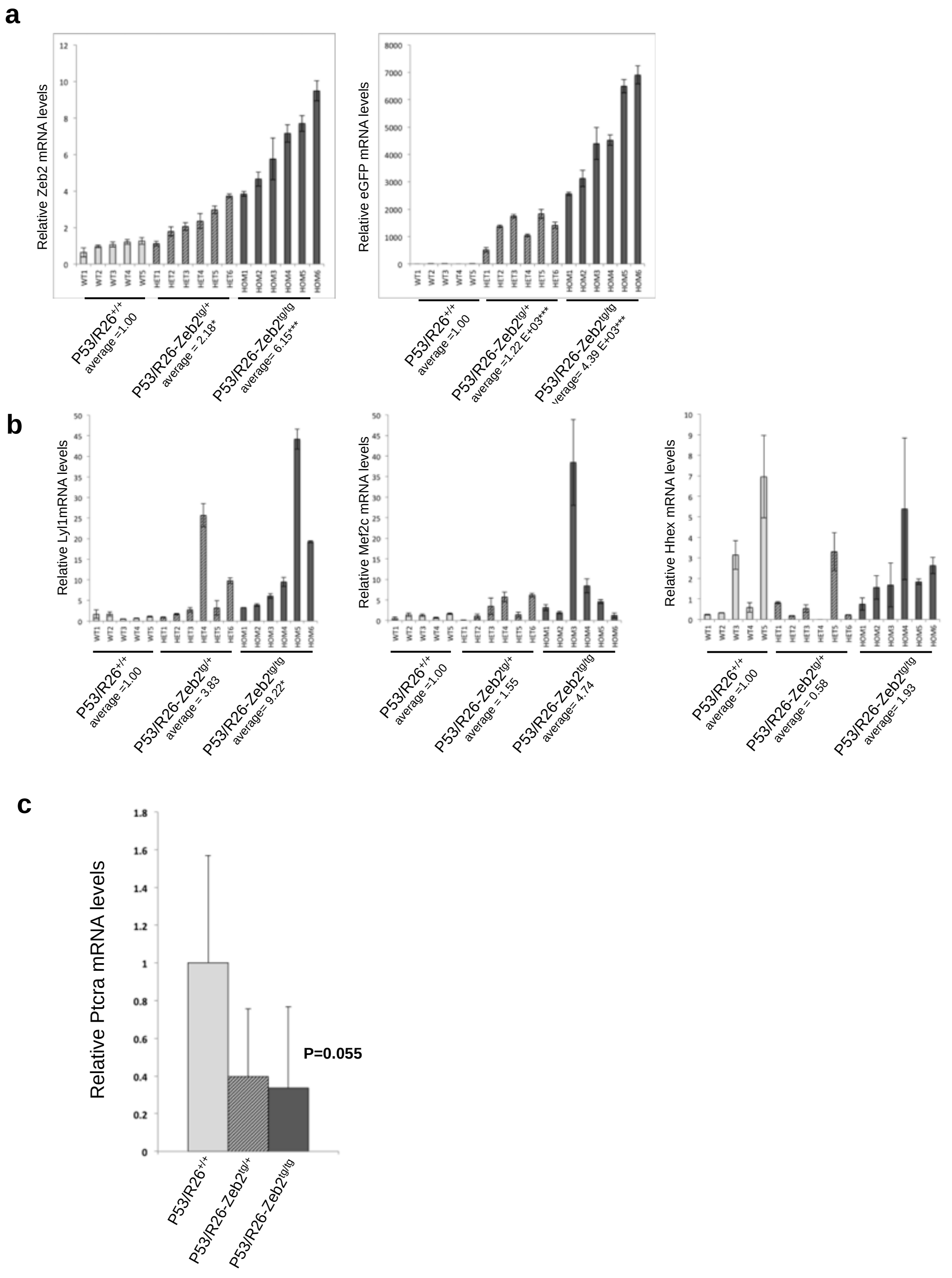

Supplementary Figure 7. Expression of immature/ETP-ALL marker genes in mouse thymic tumors upon *Zeb2* overexpression.

(a) qRT-PCR analysis for *Zeb2* and *eGFP* in control P53/ZEB2$^{+/+}$ and *Zeb2* overexpressing *P53/R26-Zeb2*$^{tg/+}$ and *P53/R26-Zeb2*$^{tg/tg}$ tumors. Relative mRNA levels are shown for each independent tumor sample, average of triplicate experiment with stdev. (technical errors) **(b)** qRT-PCR analysis for the immature/ETP-ALL marker genes *Lyl1*, *Mef2c* and *Hhex* in control *P53/ZEB2*$^{+/+}$ versus *P53/R26-Zeb2*$^{tg/+}$ and *P53/R26-Zeb2*$^{tg/tg}$ tumors. Relative

mRNA levels are shown for each independent tumor sample, average of triplicate experiment with stdev (technical errors). Below each group of tumors, mean expression level is indicated relative to control group and significant differences. One-way ANOVA was used for statistical analysis. **(c)** qRT-PCR analysis for *Ptcra* in control $P53/ZEB2^{+/+}$ and $P53/R26\text{-}Zeb2^{tg/+}$ or $P53/R26\text{-}Zeb2^{tg/tg}$ tumors (n=3/genotype).
* P<0.05, ** P<0.01, *** P<0.001 (versus control)

a

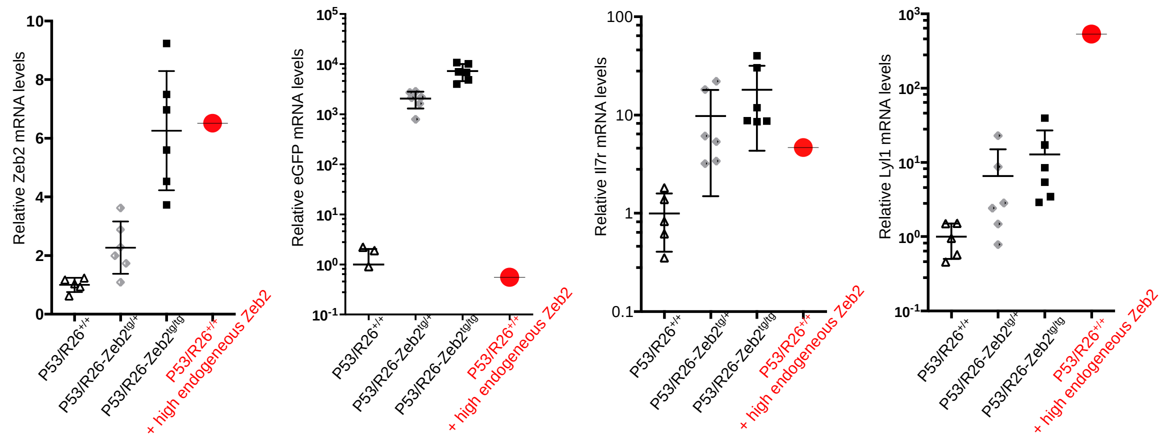

b

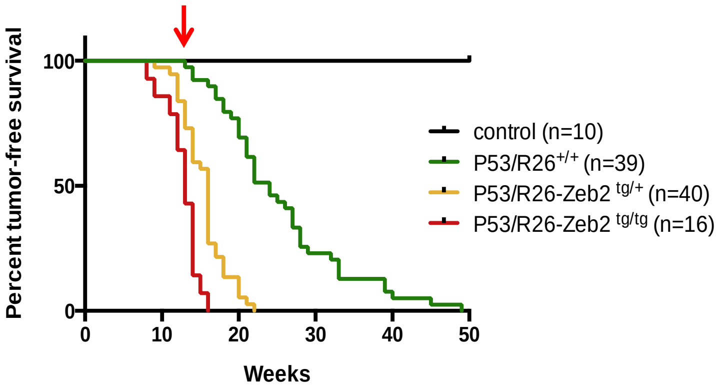

Supplementary Figure 8. Tie2cre, p53$^{fl/fl}$ thymic tumor with spontaneous high *Zeb2* mRNA levels

(a) Real time qRT-PCR for *Zeb2*, *eGFP*, *Il7r* and the immature marker gene *Lyl1* in multiple control and *Zeb2* overexpressing thymic tumors. One of the control tumors (indicated in each panel in red) showed high endogenous *Zeb2* mRNA levels, similar to the R26-*Zeb2* overexpressing tumors, had similar high mRNA levels of *Il7r* and *Lyl1*. Mean expression is indicated with stdev. **(b)** Kaplan-Meier survival curves showing tumor latency of this control sample with high Zeb2 levels (red arrow) was lower than the rest of the control group, and was more similar to the tumor development in the Zeb2 overexpressing mice.

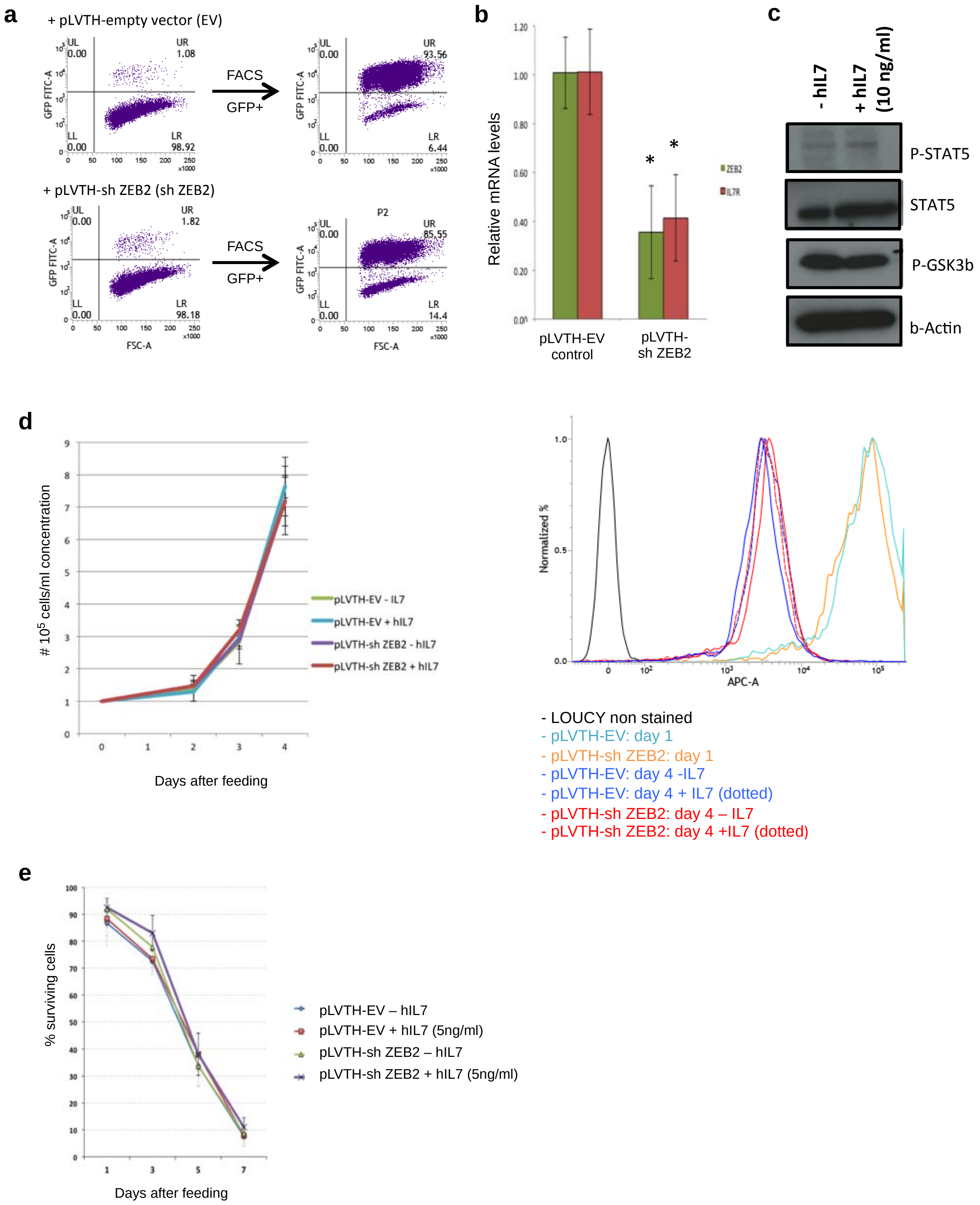

Supplementary Figure 9. ZEB2 Stable knockdown in immature LOUCY cells

(a) FACS plot showing 1-2% eGFP positive cells after transduction of LOUCY cells with either pLVTH-empty vector or pLVTH-shZEB2, and after enrichment by FACS sorting **(b)** 65% ZEB2 knockdown is correlated with a decrease of IL7R mRNA levels, as analyzed by qRT-PCR. Average expression is shown with stdev (technical replicates). Student T-test used for statistical analysis. **(c)** Western blot analysis for STAT5 and p-STAT5 after IL7

administration, indicating that LOUCY cells have lost their IL7 responsiveness. **(d)** No effects were seen on the proliferation rate of LOUCY after stabile ZEB2 knockdown analyzed via standard growth curve analysis (left) and flow cytometry (right). Histogram is showing that both cell lines (control pLVTH-EV versus pLVTH-shZEB2) were equally labeled with the Cell Proliferation Dye eFluor670 at day 1. At day 4, both cell lines (with and without IL7 administration) showed similar intense signal of the dye equally distributed between daughter cells. **(e)** Similarly, no IL7 effect before and after *ZEB2* knockdown was observed on LOUCY cell survival, analyzed by flow cytometry. * $P<0.05$

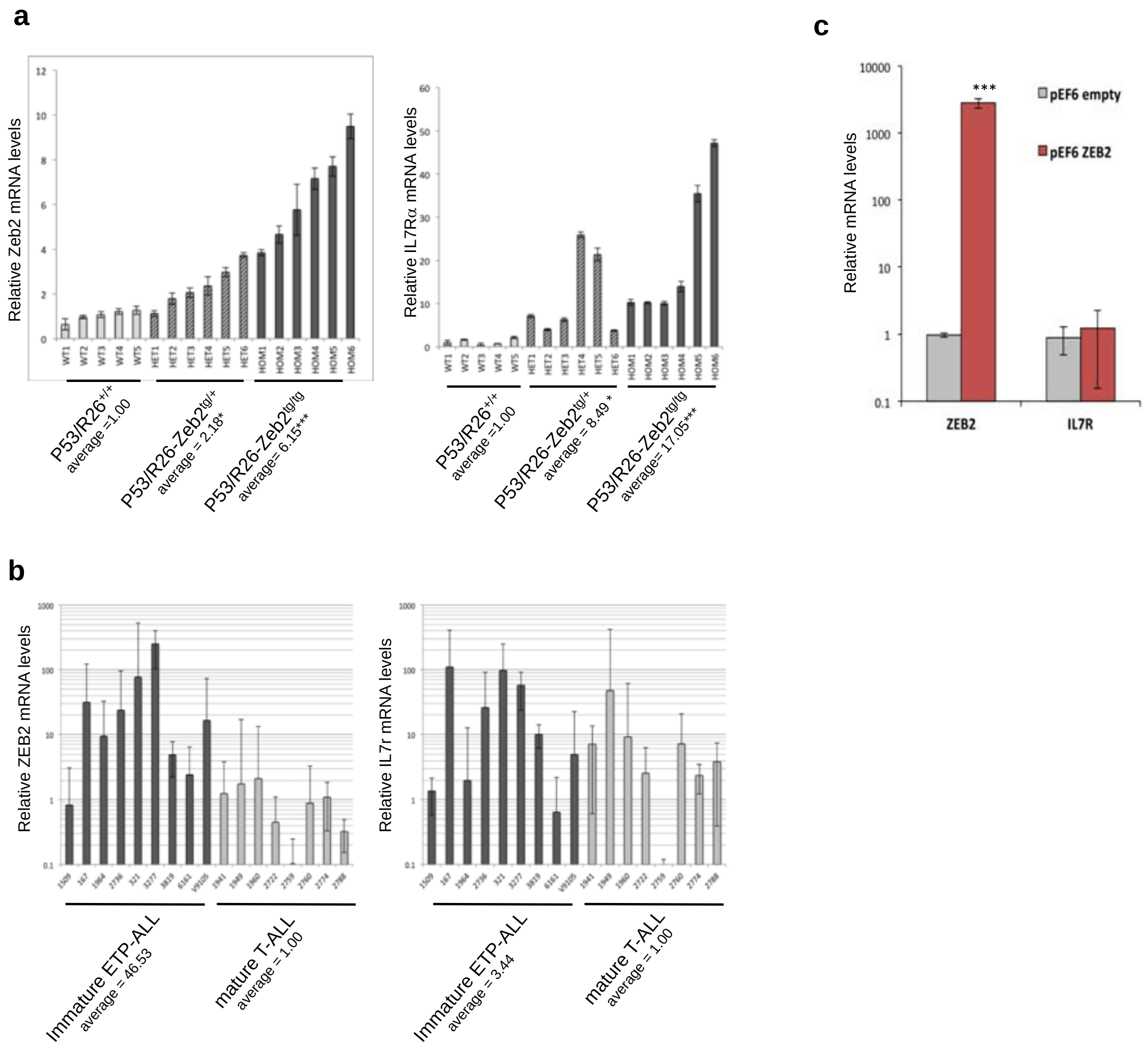

Supplementary Figure 10. *IL7R* is putative direct downstream target of ZEB2 in an immature ETP-ALL context.

(a) *Zeb2* and *Il7r* mRNA expression levels are correlated in mouse tumor samples. mRNA levels are shown for each independent tumor sample, average of triplicate experiments with Standard Deviation (technical errors). Below each group of tumors, mean expression level is indicated relative to the control group, and significant differences (one-way ANOVA) are indicated.**(b)** *ZEB2* and *IL7R* mRNA expression levels are correlated in human T-ALL patients. Patients are grouped into immature and mature subclasses based on an unbiased hierarchical clustering of their gene expression profile. Below each subclass of patients, mean expression level is indicated relative to mature T-ALL group. **(c)** qRT-PCR analysis for *ZEB2* and *IL7R* mRNA levels after transient overexpression of *ZEB2* in the mature human T-ALL cell line JURKAT, indicating that the correlated expression of *ZEB2* and *IL7R* is immature ETP/ALL context dependent. The results are the average ± stdev. of 3 independent transient transfections. * P<0.05, ** P<0.01, *** P<0.001 (versus control)

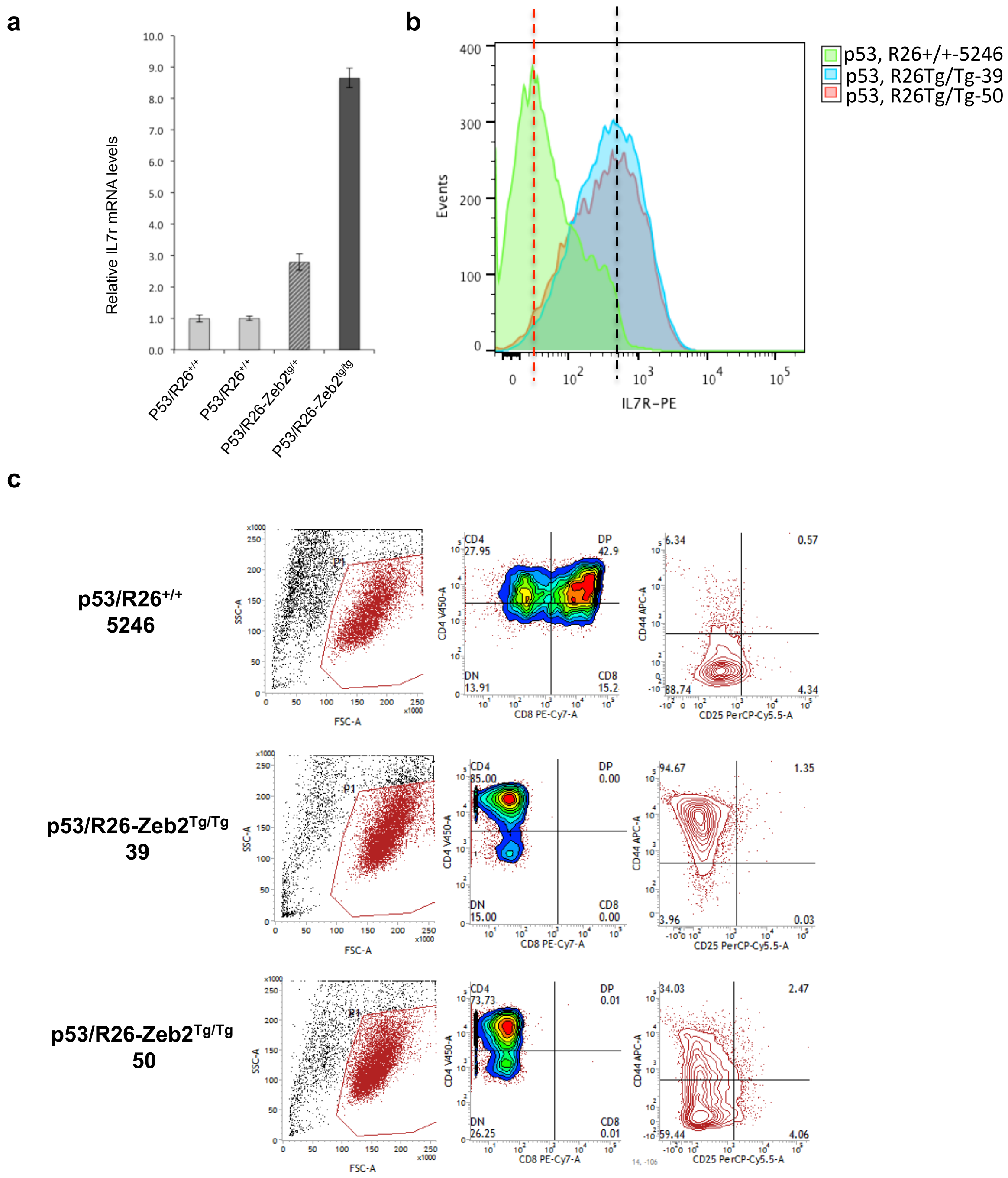

Supplementary Figure S11. Characterization of thymic tumor derived mouse cell lines **(a)** qRT-PCR analysis for *IL7r* in derived cell lines. Similar to the primary tumors, we see increased *Il7r* mRNA levels in the *Zeb2* overexpressing cell lines. Average expression is shown with stdev. (technical replicates). **(b)** Histogram showing increased IL7R protein levels in *Zeb2* overexpressing cell lines as demonstrated by flow cytometry. **(c)** Immunophenotype of the mouse cell lines as analyzed by flow cytometry. *P53/R26-Zeb2*$^{tg/tg}$ overexpressing cell lines are more immature, lack the mature marker CD8 and have more CD44+ cells compared to the more mature control *P53/R26*$^{+/+}$ cell line.

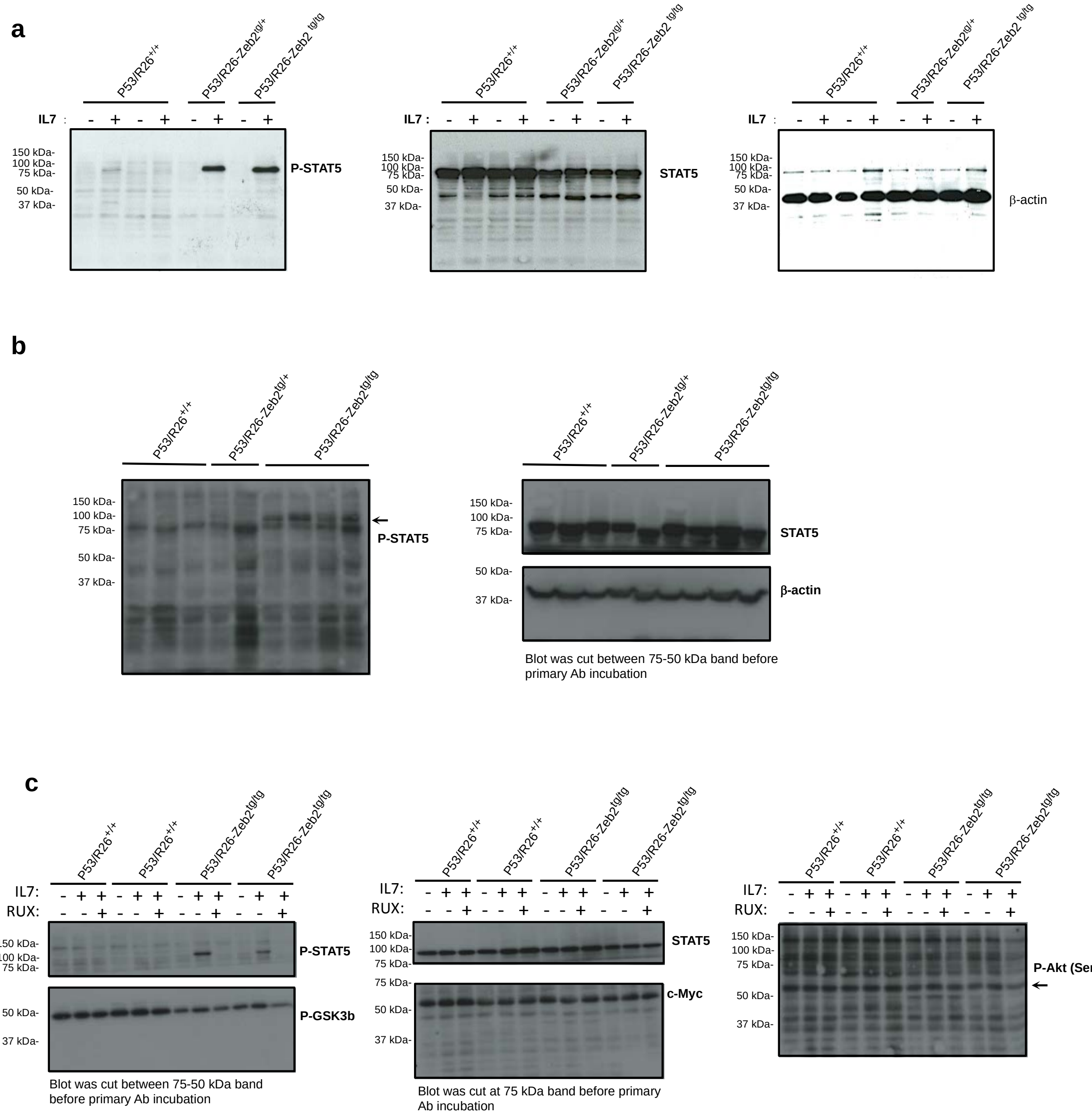

Supplementary Figure 12. Full-size scan of Western blots

(a) Full size scan of Western blot from Figure 5f. **(b)** Full size scan of Western blot from Figure 5g. **(c)** Full size scan of Western blot from Figure 6c.

Supplementary Table 1. Neoplastic/preneoplastic lesions spectrum and frequency in the different cohorts examined

**Neoplastic/preneoplastic lesions cathegories**	***P53/R26*** $^{+/+}$ **(# mice = 25)**	***P53/R26-Zeb2*** $^{tg/+}$ **(# mice = 18)**	***P53/R26-Zeb2*** $^{tg/tg}$ **(# mice = 7)**
Hematopoietic	23	20	8
ATH	2	0	0
Pre-T LBL/L	16	18	7
(thymic)	(9)	(5)	(4)
(generalized/systemic)	(7)	(13)	(3)
Uncharacterized multicentric (non thymic) T L	4	0	0
SMZL	1	1	0
ML	0	1	1
Vascular	17	0	0
HMA	2	0	0
HSA	15	0	0
(solitary)	(3)	(0)	(0)
(multicentric)	(11)	(0)	(0)
(multicentric with metastases)	(1)	(0)	(0)
Miscellaneous	2	1	0
PA	1	0	0
IPA	0	1	0
NGCO	1	0	0
Total no. of lesions	42	21	8

ATH, atypical thymic hyperplasia; Pre-T LBL/L, precursor T-cell lymphoblastic lymphoma/leukemia; T L, T cell lymphoma; SMZL, splenic marginal zone lymphoma; ML, myeloid leukemia; HMA, hemangioma; HAS, hemangiosarcoma; PA, solitary pulmonary adenoma; IPA, solitary intestinal polypoid adenoma; NGCO, solitary nongestational choriocarcinoma of the ovary

Supplementary Table 2. Overview of the activating *Notch1* mutations and loss of function mutations of the tumor suppressor genes *Pten* and *Ikzf1* found by array CGH and sequencing of R26-Zeb2$^{tg/tg}$ and as well control *P53/R26*$^{+/+}$ and Zeb2 overexpressing *P53/R26-Zeb2*$^{tg/+}$ or *P53/R26-Zeb2*$^{tg/tg}$ thymic tumors

Tumor sample	*Notch1*	*Pten*	*Ikzf1*
**R26-Zeb2tg/tg_1**	-	-	-
**R26-Zeb2tg/tg_2**	-	deletion	deletion
**R26-Zeb2tg/tg_3**	-	-	-
**P53/R26$^{+/+}$_1**	-	p.[Y155N;V158I]	-
**P53/R26$^{+/+}$_2**	-	deletion	-
**P53/R26$^{+/+}$_3**	-	deletion	-
**P53/R26$^{+/+}$_4**	-	-	-
**P53/R26$^{+/+}$_5**	-	-	-
**P53/R26$^{+/+}$_6**	-	-	-
**P53/R26-Zeb2$^{tg/+}$_1**	p.R2361*37	-	-
**P53/R26-Zeb2$^{tg/+}$_2**	p.P2459*32	-	-
**P53/R26-Zeb2$^{tg/+}$_3**	-	deletion	deletion
**P53/R26-Zeb2$^{tg/+}$_4**	-	deletion	deletion
**P53/R26-Zeb2$^{tg/+}$_5**	-	deletion	-
**P53/R26-Zeb2$^{tg/+}$_6**	-	deletion	-
**P53/R26-Zeb2$^{tg/+}$_7**	-	deletion	-
**P53/R26-Zeb2$^{tg/+}$_8**	-	-	deletion
**P53/R26-Zeb2$^{tg/+}$_9**	-	-	deletion
**P53/R26-Zeb2$^{tg/tg}$_1**	-	deletion	deletion
**P53/R26-Zeb2$^{tg/tg}$_2**	-	deletion	-
**P53/R26-Zeb2$^{tg/tg}$_3**	-	-	deletion
**P53/R26-Zeb2$^{tg/tg}$_4**	-	-	-

Supplementary Table 3. Scoring of cKit expression in control *P53/R26*$^{+/+}$ and *Zeb2* overexpressing *P53/R26-Zeb2*$^{tg/+}$ or *P53/R26-Zeb2*$^{tg/tg}$ thymic tumors via immunohistochemistry

Genotype	Intensity of cKit (0-3)	Distribution of cKit (0-3)	Combined score (0-6)
**P53/R26$^{+/+}$_1**	2	3	5
**P53/R26$^{+/+}$_2**	0	0	0
**P53/R26$^{+/+}$_3**	2	3	5
**P53/R26$^{+/+}$_4**	0	0	0
**P53/R26$^{+/+}$_5**	1	1	2
**P53/R26$^{+/+}$_6**	1	1	2
**P53/R26$^{+/+}$_7**	0	0	0
**P53/R26$^{+/+}$_8**	1	1	2
**P53/R26$^{+/+}$_9**	1	1	2
**P53/R26$^{+/+}$_10**	0	0	0
**P53/R26$^{+/+}$_11**	1	1	2
**P53/R26$^{+/+}$_12**	1	1	2
**P53/R26$^{+/+}$_13**	3	1	4
**P53/R26$^{+/+}$_14**	2	1	3
**P53/R26-Zeb2$^{tg/+}$_1**	2	3	5
**P53/R26-Zeb2$^{tg/+}$_2**	3	3	6
**P53/R26-Zeb2$^{tg/+}$_3**	2	3	5
**P53/R26-Zeb2$^{tg/+}$_4**	1	2	3
**P53/R26-Zeb2$^{tg/+}$_5**	2	3	5
**P53/R26-Zeb2$^{tg/+}$_6**	1	2	3
**P53/R26-Zeb2$^{tg/+}$_7**	2	3	5
**P53/R26-Zeb2$^{tg/+}$_8**	3	3	6
**P53/R26-Zeb2$^{tg/+}$_9**	2	3	5
**P53/R26-Zeb2$^{tg/+}$_10**	1	2	3
**P53/R26-Zeb2$^{tg/+}$_11**	2	1	3
**P53/R26-Zeb2$^{tg/+}$_12**	3	2	6
**P53/R26-Zeb2$^{tg/tg}$_1**	2	2	4
**P53/R26-Zeb2$^{tg/tg}$_2**	1	2	3
**P53/R26-Zeb2$^{tg/tg}$_3**	3	3	6
**P53/R26-Zeb2$^{tg/tg}$_4**	1	3	4
**P53/R26-Zeb2$^{tg/tg}$_5**	3	2	5
**P53/R26-Zeb2$^{tg/tg}$_6**	3	3	6

Intensity and distribution cKit immunoreactivity in the lymphomatous compartment was semiquantitatively scored as followed:

- Intensity of immunoreactivity:

  0= no immunoreactivity

  1= weak membranous to cytoplasmic immunoreactivity

  2= moderate membranous to cytoplasmic immunoreactivity

  3= marked membranous to cytoplasmic immunoreactivity

- Distribution of immunoreactivity

  0 = no immunoreactivity

  1 = immunoreactive lymphomatous cells accounting for less than 20% of the entire tumor cell population

  2 = immunoreactive lymphomatous cells comprised between 20% and 50% of the entire tumor cell population

  3 = immunoreactive lymphomatous cells accounting for more than 50% of the entire tumor cell population

Supplementary Table 4. Details concerning primary antibodies and procedures used for the immunohistochemical examination

Antigen	Primary antibody	Source	Antigen retrieval	Working dilution	Incubation time	Detection system	Staining method
CD31 (PECAM)	SZ31 Rat mon[a]	Dianova	HIER[c]	1:50	1h RT[d]	ABC[e]	automated[g]
CD45/CLA	30-F11 Rat mon	BD-Pharmigen	HIER	1:150	1h RT	ABC	manual
CD3	A0452 Rb Poly[b]	Dako	HIER	1:150	1h RT	EnVision[f]	automated
CD45R/B220	RA3-6B2 Rat mon	BD-Pharmigen	HIER	1:150	1h RT	ABC	automated
cKit	A4502 Rb Poly	Dako	HIER	1:400	1h RT	ABC	automated
CD20	#RB-9013 Rb Poly	LabVision	HIER	1:50	1h RT	EnVision	manual
Lyve1	ab14917 Rb poly	Abcam	HIER	1:100	1h RT	ABC	manual
IBA-1	019-19741 Rb poly	Wako	HIER	1:1000	1h RT	ABC	manual

[a]rat monoclonal; [b]rabbit polyclonal; [c]heat-induced epitope retrival, 2100 (Retriever, PickCell Labs, Lelystad, The Netherlands), 0.01 mol/L citrate buffer pH = 6.0 (Diapath SpA, Martinengo, Bergamo, Italy); [d]one hour at room temperature; [e]avidin-biotin complex (Vectastain Elite ABC kit PK-6100, Vector Laboratories Inc., Burlingame, CA, USA); [f]Dako EnVision[TM] system (DakoCytomation, Glostrup, Denmark); [g]Thermo Scientific, LabVision Autostainer 480S

Supplementary Table 5. Antibodies used for FACS and flow cytometry

Antigen	Conjugated	dilution	Company	Experiment
Lineage: Gr-1	Biotin	2μl;10^6 cells	eBioscience	FACS sort
Lineage: CD3e	Biotin	2μl;10^6 cells	eBioscience	FACS sort
Lineage: B220	Biotin	2μl;10^6 cells	eBioscience	FACS sort
Lineage: Ter119	Biotin	2μl;10^6 cells	eBioscience	FACS sort
Streptavidin	PE	1;500	BD Bioscience	FACS sort
cKit/CD117	APC	1;200	Immunoscource	FACS sort + flow cytometry
cKit/CD117	APC.H7	1;100	BD Bioscience	Flow cytometry
CD4	Biotin	1;200	BD Bioscience	Flow cytometry
CD8a	PE.Cy7	1;100	eBioscience	Flow cytometry
CD3e	V500	1;100	BD Bioscience	Flow cytometry
CD3e	PE.Cy7	1;100	eBioscience	Flow cytometry
CD25	PerCP.Cy5.5	1;100	BD Bioscience	Flow cytometry
CD44	APC	1;100	eBioscience	Flow cytometry
Thy1/CD90	FITC	1;100	BD Bioscience	Flow cytometry
Streptavidin	V500	1;200	BD Bioscience	Flow cytometry
Streptavidin	eFluor780	1;200	eBioscience	Flow cytometry
IL7R	PE	1;100	Biolegend	Flow cytometry

Supplementary Table 6. Primers sequences used

GENE	forward primer	reverse primer	experiment
**□otch1 EX26**	5’-ACGGGAGGACCTAACCAAAC-3’	5’-CAGCTTGGTCTCCAACACCT-3’	Mutation screen
**□otch1 EX27**	5’-CGCTGAGTGCTAAACACTGG-3’	5’-GTTTTGCCTGCATGTACGTC-3’	Mutation screen
**Notch1 EX34-1**	5’-GCTCCCTCATGTACCTCCTG-3’	5’-TAGTGGCCCCATCATGCTAT-3’	Mutation screen
**Notch1 EX34-2**	5’-ATAGCATGATGGGGCCACTA-3’	5’-CTTCACCCTGACCAGGAAAA-3’	Mutation screen
**Notch1 EX34-3**	5’-GTAGCCGGTTGGCCTTTG-3’	5’-GCTTTCCTGGGGCAGAAT-3’	Mutation screen
**Notch1 EX34-4**	5’-TGAGCCTGGTGGTCTAGGAT-3’	5’-CCTGTGTGGCAGACTTGAGA-3’	Mutation screen
**Pten EX5**	5’-GGCTTCTTTTAAGAACCAGTAAGTT-3’	5’-CACAAAGAGGGAGGAAGGAA-3’	Mutation screen
**Pten EX7**	5’-TGATAAGTTGAAGACATTTCTTGTGA-3’	5’-CCAAAGGCTTTAAGCAAAAGG-3’	Mutation screen
**□-actin**	5'-AGTGTGACGTTGACATCCGTA-3’	5’-GCCAGAGCAGTAATCTCCTTCT-3’	qRTPCR
**Gapdh**	5’-AGGTTGTCTCCTGCGACTTCA-3’	5’-GGTGGTCCAGGGTTTCTTACTC-3’	qRT-PCR
**Rpl13**	5’-CCTGCTGCTCTCAAGGTTGTT-3’	5’-TGGTTGTCACTGCCTGGTACTT-3’	qRT-PCR
**Tbp**	5’-TCTACCGTGAATCTTGGCTGTAAA-3’	5’-TTCTCATGATGACTGCAGCAAA-3’	qRT-PCR
**eGFP**	5’-CAAGCAGAAGAACGGCATCA-3’	5’-AGGTAGTGGTTGTCGGGCA-3’	qRT-PCR
**Zeb2**	5’-AGCGACACGGCCATTATTTAC-3’	5’-GTTGGGCAAAAGCATCTGGAG-3’	qRT-PCR
**Lyl1**	5’-CCAGTGATAAACCTGGGACAC-3’	5’-CTGCTGGCCCAATGTAGACA-3’	qRT-PCR
**Hhex**	5’-CGGACGGTGAACGACTACAC-3’	5’-CGTTGGAGAACCTCACTTGAC-3’	qRT-PCR
**Mef2c**	5’-AGATACCCACAACACACCACGCGCC-3’	5’-ATCCTTCAGAGAGTCGCATGCGCTT	qRT-PCR
**Il7r**	5’-GCGGACGATCACTCCTTCTG-3’	5’-AGCCCCACATATTTGAAATTCCA-3’	qRT-PCR
**ZEB2**	5’-ATCAGATGAGCTTCCTACCA-3’	5’-GCAATTCTCCCTGAAATCCT-3’	qRT-PCR
**LYL1**	5’-GAAGAAGGACCAGTGAAGAC-3’	5’-AGAGGGTTGTGGGTGATTT-3’	qRT-PCR
**HHEX**	5’-ATCGACGCGCTAAATG-3’	5’-ATGCCAATGCCAGTGG-3’	qRT-PCR
**MEF2C**	5’-GGACTTGGATTGCTTCAGAT-3’	5’-ATGCTTGTATGCCCTCATTT-3’	qRT-PCR
**PSCD4**	5’-GCACGGGTCATCTTTTC-3’	5’-CTTGCGCCCAATACAC-3’	qRT-PCR
**FAM64A**	5’-ACTGCCTGTTGGACTTCTT-3’	5’-TTTGCCACTGTTGTTTGAC-3’	qRT-PCR
**IL7R**	5’-GGAGAAAGTGGCTATGCTCAAAA-3’	5’-TCCATTCACTTCCAACTGGCTAT-3’	qRT-PCR
**IL7R promoter**	5’-TCCGCACTCTATTTAGATTTC-3’	5’-TCATTTAAGTGGACCATCATTT-3’	ChIP
**Actin**	5’-TGGGCCGTTAGCTAGTGTCT-3’	5’-CAGCTGTGGCTGCACATAAT-3’	ChIP

Supplementary References

1. Soulier, J., *et al.* HOXA genes are included in genetic and biologic networks defining human acute T-cell leukemia (T-ALL). *Blood* 106, 274-286 (2005).
2. Van Vlierberghe, P., *et al.* ETV6 mutations in early immature human T cell leukemias. *The Journal of experimental medicine* 208, 2571-2579 (2011).
